# Enzymatic comparison of two homologous enzymes reveals N-terminal domain of chondroitinase ABC I regulates substrate selection and product generation

**DOI:** 10.1016/j.jbc.2023.104692

**Published:** 2023-04-07

**Authors:** Min Du, Lin Wei, Min Yuan, Ruyi Zou, Yingying Xu, Xu Wang, Wenshuang Wang, Fuchuan Li

**Affiliations:** 1 National Glycoengineering Research Center and Shandong Provincial Key Laboratory of Carbohydrate Chemistry and Glycobiology, Shandong University, Qingdao, China; 2 College of Marine Life Sciences, Ocean University of China, Qingdao, China

**Keywords:** glycosaminoglycan, chondroitin sulfate, dermatan sulfate, lyase, CSase ABC, enzymatic comparison, substrate selection, product generation

## Abstract

Chondroitinase ABC-type I (CSase ABC I), which can digest both chondroitin sulfate (CS) and dermatan sulfate (DS) in an endolytic manner, is an essential tool in structural and functional studies of CS/DS. Although a few CSase ABC I have been identified from bacteria, the substrate-degrading pattern and regulatory mechanisms of them have rarely been investigated. Herein, two CSase ABC I, IM3796 and IM1634, were identified from the intestinal metagenome of CS-fed mice. They show high sequence homology (query coverage: 88.00%, percent identity: 90.10%) except for an extra peptide (Met^1^–His^109^) at the N-terminus in IM1634, but their enzymatic properties are very different. IM3796 prefers to degrade 6-*O*-sulfated GalNAc residue-enriched CS into tetra- and disaccharides. In contrast, IM1634 exhibits nearly a thousand times more activity than IM3796 and can completely digest CS/DS with various sulfation patterns to produce disaccharides, unlike most CSase ABC I. Structure modeling showed that IM3796 did not contain an N-terminal domain composed of two β-sheets, which is found in IM1634 and other CSase ABC I. Furthermore, deletion of the N-terminal domain (Met^1^–His^109^) from IM1634 caused the enzymatic properties of the variant IM1634-T109 to be similar to those of IM3796, and conversely, grafting this domain to IM3796 increased the similarity of the variant IM3796-A109 to IM1634. In conclusion, the comparative study of the new CSase ABC I provides two unique tools for CS/DS-related studies and applications and, more importantly, reveals the critical role of the N-terminal domain in regulating the substrate binding and degradation of these enzymes.

Chondroitin sulfate/dermatan sulfate (CS/DS) is a subset of structurally complex acidic linear polysaccharides that are classified into glycosaminoglycans (GAGs); is a side chain of proteoglycans (PGs) on cell surfaces and in the extracellular matrix (ECM) (1); and is involved in many physiological phenomena, such as the development of the central nervous system (2, 3), cytokinesis (1), cell division (4), growth factor signaling (5, 6), morphogenesis (7), and inflammation (8, 9). The various functions of CS/DS are due to their heterogeneous structures (10). In the biosynthesis of CS/DS, a linkage tetrasaccharide (glucuronic acid-galactose-galactose-xylose-*O*-Ser) is first synthesized onto a specific serine residue of core protein CS/DSPGs, and subsequently, D-glucuronic acid (GlcA) or *N*-acetyl-D-galactosamine (GalNAc) residues are alternately transferred to the linkage by particular glycosyltransferases to form the precursor backbone (-4GlcAβ1-3GalNAcβ1-) of CS (11, 12, 13). Then, the CS backbone is modified through the isomerization of GlcA to the IdoA residue to form the DS backbone (-4IdoAα1-3GalNAcβ1-) by the catalysis of C5-epimerase (14, 15) and sulfation of C-2 of GlcA/IdoA and/or C-4 and/or C-6 of GalNAc by several sulfotransferases (16, 17, 18). Thus, CS and DS are usually present in the hybrid form of CS-DS in animal tissues (19, 20). In addition, CSs are usually named according to the main disaccharide units or the rare disaccharide units they contain, such as CS-A, which mainly contains GlcAβ1-3GalNAc(4S) (A unit); CS-C, which mainly contains GlcAβ1-3GalNAc(6S) (C unit); CS-D, which is rich in GlcA(2S)β1-3GalNAc(6S) (D unit); and CS-E, which is rich in GlcAβ1-3GalNAc(4S,6S) (E unit). These modifications lead to the high structural complexity of CS/DS chains, which endows CS/DS with a variety of biological roles but introduces great difficulties to the structural and functional study of CS/DS.

Bacteria-derived CS/DS lyases can cleave the *β*1-4 glucosidic bond between GalNAc and GlcA/IdoA to yield a double bond between C4 and C5 of hexuronic acid *via* a *β*-elimination mechanism, which is essential in structural and functional studies of CS/DS (21, 22). Based on their substrate specificity, CS/DS lyases can be divided into the following major categories: chondroitinase (CSase) ABC, which can digest CS/DS as well as hyaluronic acid (HA) (23); CSase AC, which selectively cleaves CS and HA (24); CSase C, which prefers to act upon C unit-rich domains in the CS/DS chain (25); and CSase B, which is specific for DS (26). Among them, CSase ABCs are important tools in the structural and functional studies of CS/DS (27, 28, 29, 30, 31, 32), while only a few CSase ABCs have been identified from *Proteus vulgaris*, *Bacteroides stercoris*, *Flavobacterium heparium*, *Photobacterium* sp., and *Vibrio* sp (33, 34, 35, 36, 37). In addition, it has been found that CS/DS lyases in the same category can produce final products with different molecular sizes when the same substrate is digested. For example, the final digests of CS/DS by CSase ABC I from *P. vulgaris* (ATCC6896) (33) or enCSase from *Photobacterium* sp. are tetrasaccharides and disaccharides (35) while those by CSase ABC from *Vibrio* sp. QY108 are disaccharides (37). However, the molecular mechanisms involved in the enzymatic properties of CS/DS lyases have rarely been investigated despite their great importance for the application and engineering of these enzymes.

In the present study, two CSase ABC I-type enzymes (CSase IM3796 and CSase IM1634) belonging to the polysaccharide lyase 8 (PL8) family were identified from the intestinal metagenome of Kunming (KM) mice fed CS-A. Interestingly, the two enzymes share a quite high similarity of amino acid (aa) sequences (query coverage: 88.00%, percent identity: 90.10%) but show very different enzymatic properties, such as substrate specificity and product generation. In particular, IM1634 contains an additional N-terminal sequence (Met^1^–His^109^) compared with IM3796, and the deletion from IM1634 or addition to IM3796 of the additional sequence could cause the obvious interconversion of their enzymatic properties. This study should be very helpful for understanding the molecular basis that affects the enzymatic properties of CSase ABC I-type enzymes and for corresponding engineering modifications of such enzymes.

## Results

### Gene mining and sequence analysis of IM3796 and IM1634

**Figure 1:**
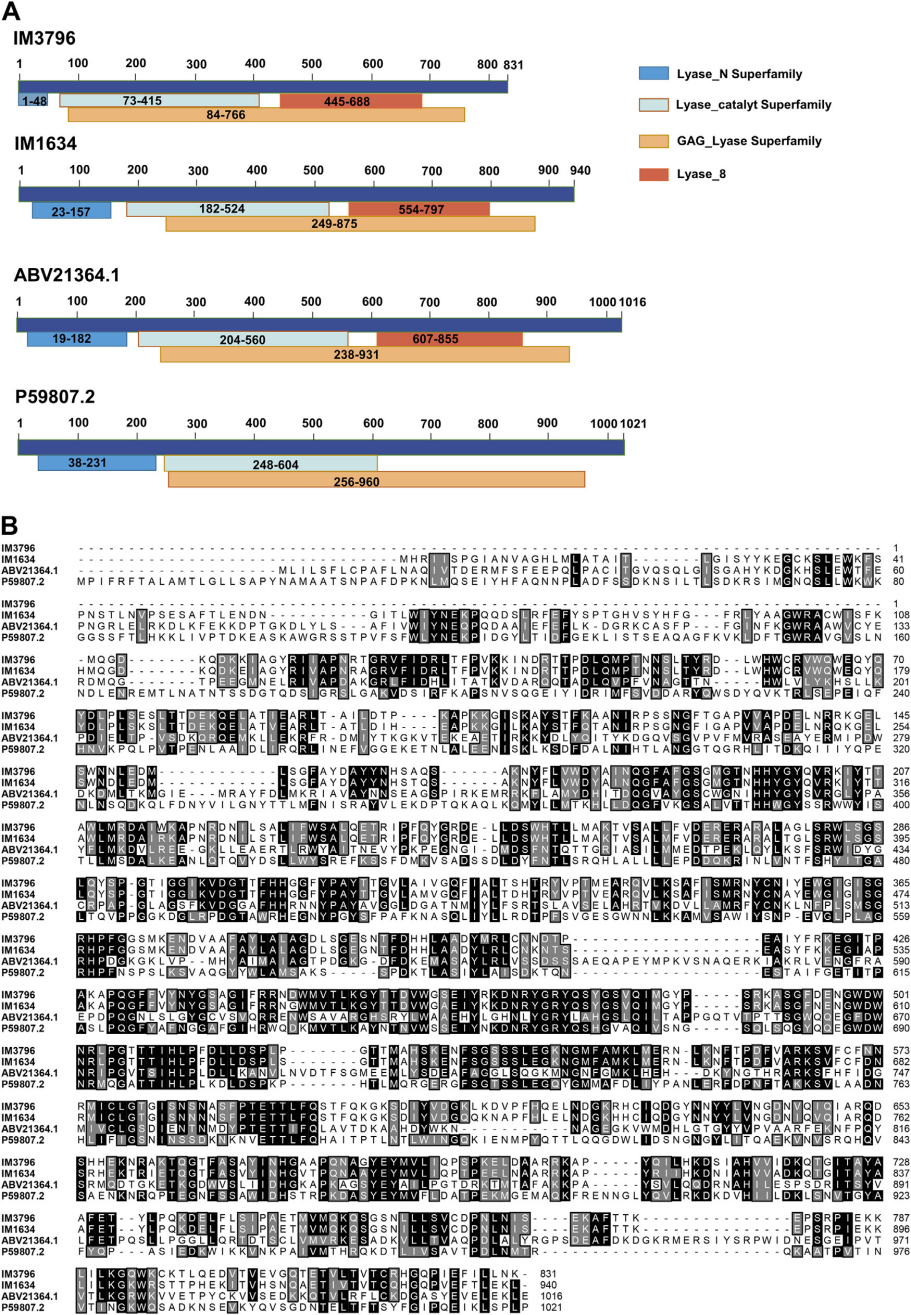
**Sequence analysis of the IM3796 and IM1634.***A*, module composition of IM3796, IM1634, CSase ABC I from *Proteus vulgaris* (GenBank accession number: P59807.2) (40), CSase ABC I from *Bacteroides thetaiotaomicron* (GenBank accession number: ABV21364.1) (54). The GAG lyase module is a hypothetical catalytic domain (Lys^84^-Pro^766^ in IM3796, Arg^249^-Pro^875^ in IM1634, Ala^256^-Pro^960^ in P59807.2 and Glu ^238^-Pro^931^ in ABV21364.1), and the module Lyase_8 (Arg^445^-Leu^688^ in IM3796, Arg^554^-Leu^797^ in IM1634 and Val^607^-Asp^855^ in ABV21364.1) is relatively conserved in PL8 family. The numbers in the graph represent the number of amino acids. *B*, multiple sequence alignment of IM3796, IM1634, and two identified CSase ABC Is from *Proteus vulgaris* (GenBank accession number: P59807.2) and *Bacteroides thetaiotaomicron* (GenBank accession number: ABV21364.1).

**Figure 2:**
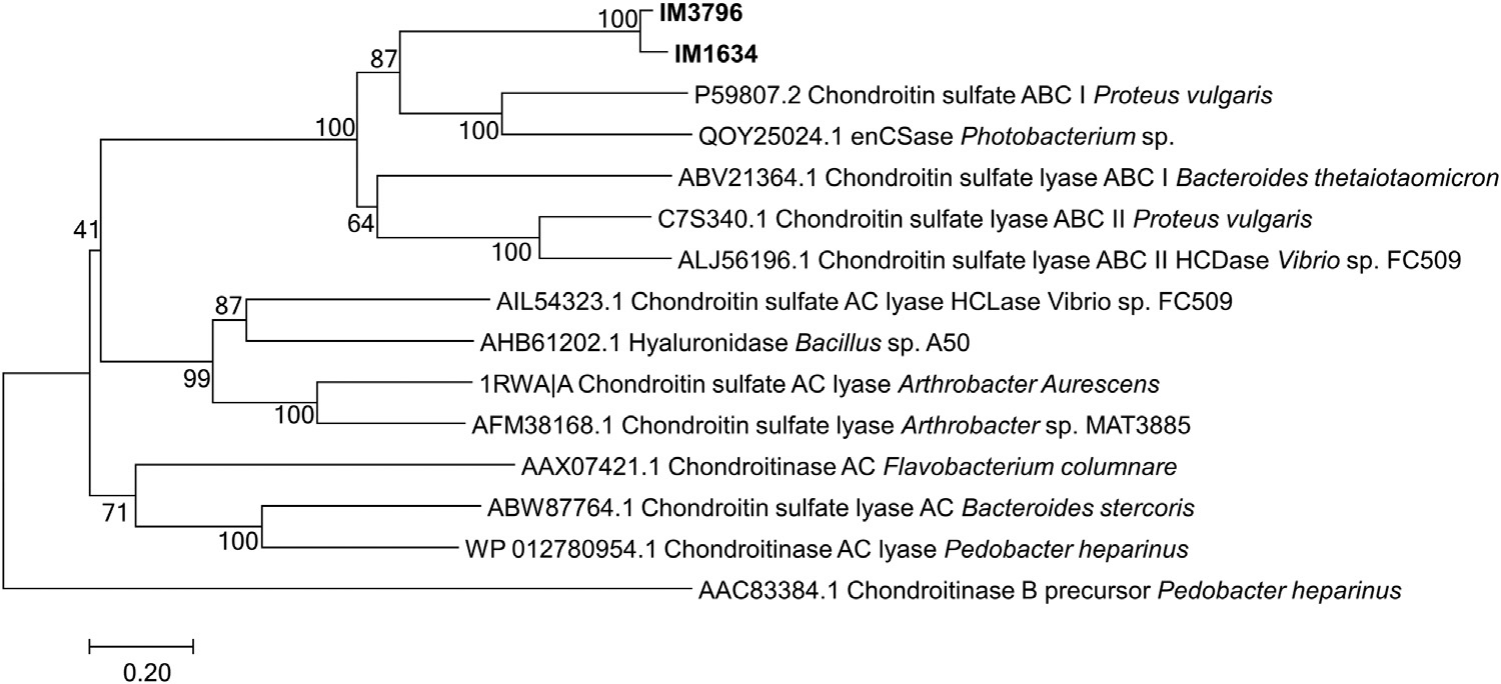
**Phylogenetic analysis of IM3796 and IM1634.** Phylogenetic analysis of IM3796 and IM1634 was executed based on Clustal W multiple alignments with identified CSases from bacteria by using the neighbor-joining method in MEGA version 7.0.26. The numbers on the branches are the bootstrap confidence values obtained from 1000 replications.

Microbial communities rich in GAG-degrading enzymes were obtained from the feces of CS-A-fed KM mice. Pairwise alignment of assembled meta-genome data against the CAZy database revealed that the metagenome of the mouse fecal microbial community encoded a broad array of potential CS/DS-degrading enzymes belonging to the PL8 family (38). Among them, the *IM3796* gene is 2496 bp in length, has a GC content of 47.4%, and encodes the 832-aa protein IM3796 with a theoretical molecular mass of 93.94 kDa and an isoelectric point (pI) of 8.81. The putative gene *IM1634* is 2823 bp in length with a GC content of 50.0%, which encodes the 941-aa protein IM1634 with a theoretical molecular mass of 106.68 kDa and a pI of 8.63. According to the sequence analysis by BLASTp, both IM3796 and IM1634 contain a “Lyase_8 module” (Arg^445^–Leu^688^ of IM3796 and Arg^554^–Leu^797^ of IM1634) (Fig. 1*A*), indicating that they belong to the PL8 family. Meanwhile, both two proteins have a “GAG_lyase superfamily module” (Lys^84^–Pro^766^ of IM3796 and Arg^249^–Pro^875^ of IM1634) like the case of two identified CSase ABC Is from *Bacteroides thetaiotaomicron* and *P. vulgaris* (Fig. 1*A*), further indicating that they should have GAG-degrading activity. Moreover, phylogenetic analysis revealed that IM3796 and IM1634 clustered with the CSase ABCs (Fig. 2), and they showed the highest amino acid identity with the CSase ABC I (P59807.2) from *P. vulgaris* (30.56%) and the CSase ABC I (ABV21364.1) from *B. thetaiotaomicron* (30.54%), respectively (Fig. 1*B*). Notably, the two enzymes are highly homologous in sequence (query coverage: 88.00%, percent identity: 90.10%), and the most significant difference between them is that IM1634 contains an additional N-terminal 109 amino acid sequence (Met^1^–His^109^) (Fig. S1).

### Cloning, expression, and purification of recombinant enzymes

**Figure 3:**
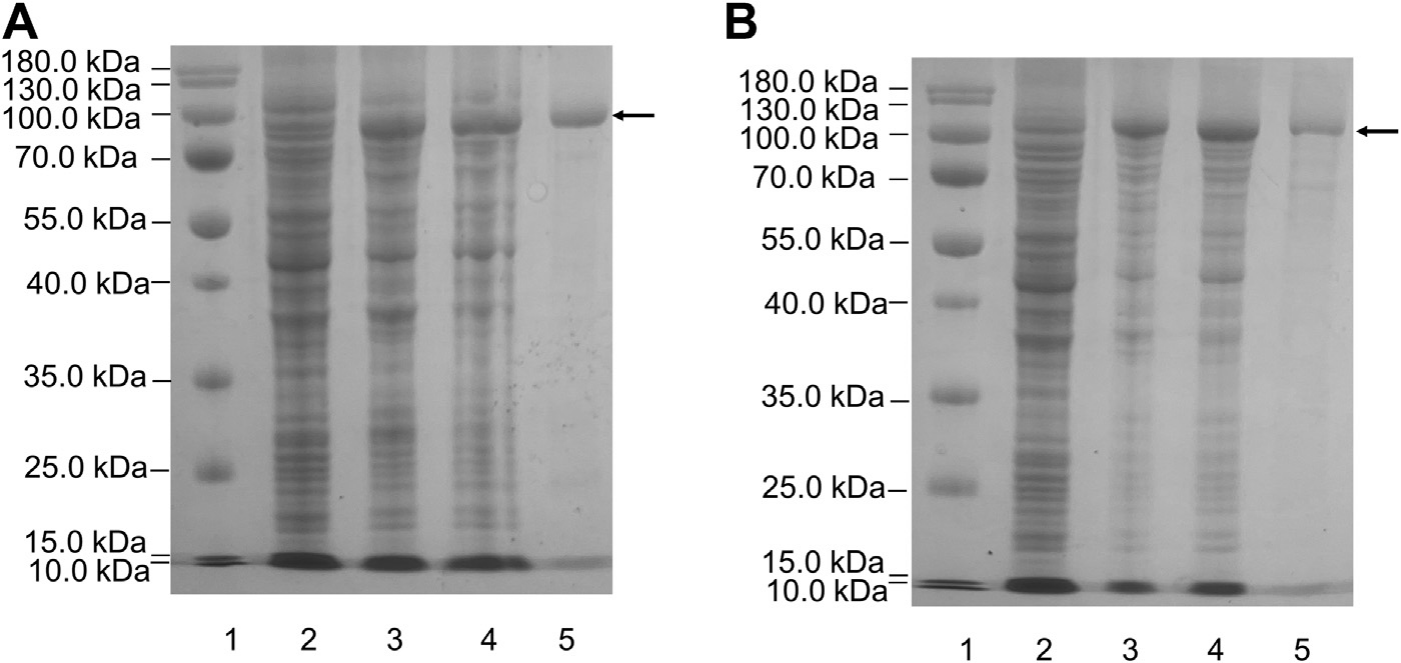
**Purification of recombinant IM3796 and IM1634 by Ni**_**2+**_**affinity chromatography.** The expression and purification of recombinant IM3796 (*A*) and IM1634 (*B*) were assessed by SDS-PAGE using 13.2% polyacrylamide gels. Lane 1, prestained protein molecular weight marker PageRuler (26616, Thermo Fisher Scientific); lane 2, lysate of cells transfected with empty plasmid (pET30a); lane 3, lysate of cells transfected with expression plasmid; lane 4, supernatant from the lysate of cells transfected with expression plasmid; and lane 5, purified recombinant IM3796 or IM1634. Molecular weight markers and their corresponding masses are also indicated.

The *IM3796* and *IM1634* genes were directly amplified from metagenomic DNA, cloned into the pET30a (+) vector, and heterologously expressed in *Escherichia coli* (*E. coli*) BL21 (DE3) cells (Table 1). The results from SDS‒PAGE analysis showed that *E. coli* BL21 (DE3) cells harboring pET30a-*IM3796* or pET30a-*IM1634* successfully expressed soluble products (approximately 240 mg/l and 300 mg/l, respectively) with the correct molecular mass. After sonication and centrifugation, the recombinant proteins in the supernatants of host cells could be purified on a Ni-NTA affinity column eluted with a gradient of imidazole. As shown in Figure 3, both IM3796 and IM1634 exhibited purities higher than 95% after affinity chromatography.

**Table 1:** Bacterial strains, plasmids, and primers used for sequencing in the present study

Materials	Description	Source
**Strains**		
*E. coli* BL21(DE3)	*F*^*−*^, *ompT*, *hsd*S (*rB*^*−*^, *mB*^*−*^), *gal*, *dcm* (DE3)	Vazyme
**Plasmid**		
pET-30a	Expression vector; kanamycin-resistant	Novagen
pET30a-IM3796	pET30a carrying a fragment encoding the recombinant protein of IM3796 fused with a His_6_ tag at the C-terminus	This study
pET30a-IM1634	pET30a carrying a fragment encoding the recombinant protein of IM1634 fused with a His_6_ tag at the C terminus	This study
Primers		
IM3796 -F	5′-ggagatatacatATGCAAGGAGACAAACAAGATAA -3′	
IM3796 -R	5′-gtggtggtgctcgagTTTGTTCAGCAGGATAAATT -3′	
IM1634-F	5′-ggagatatacatATGCACCGTATTATTAGTCCT-3′	
IM1634-R	5′-gtggtggtgctcgagCAGTTTTTCCAGGGTAAATT-3′	
IM1634-T109-F	5′-ggagatatacatatgATGCAGGGCGATAAACAGGATAA-3′	
IM1634-T109-R	5′-gtggtggtgctcgagCAGTTTTTCCAGGGTAAATTCAAC-3′	
IM3796-A109-F1	5′- ggagatatacatATGCACCGTATTATTAGTCCT-3′	
IM3796-A109-R1	5′- TTGTTTGTCTCCTTGCATGTGTTTAAAGCTAATCCA-3′	
IM3796-A109-F2	5′- TGGATTAGCTTTAAACAC ATGCAAGGAGACAAACAA-3′	
IM3796-A109-R2	5′- gtggtggtgctcgagTTTGTTCAGCAGGATAAATTCTATAGGC-3′

### Biochemical characterization of IM3796 and IM1634

First, the substrate specificities of IM3796 and IM1634 were investigated by using various GAGs and analogs. The results showed that both enzymes could degrade HA, various CSs, and DS with various sulfation patterns (Table S1), causing a significantly increasing absorbance at 232 nm but not heparan sulfate (HS), heparin (Hep), and alginate (Fig. S2), confirming that both enzymes belong to CSase ABCs and exhibit specificity to HA and CS/DS.

**Figure 4:**
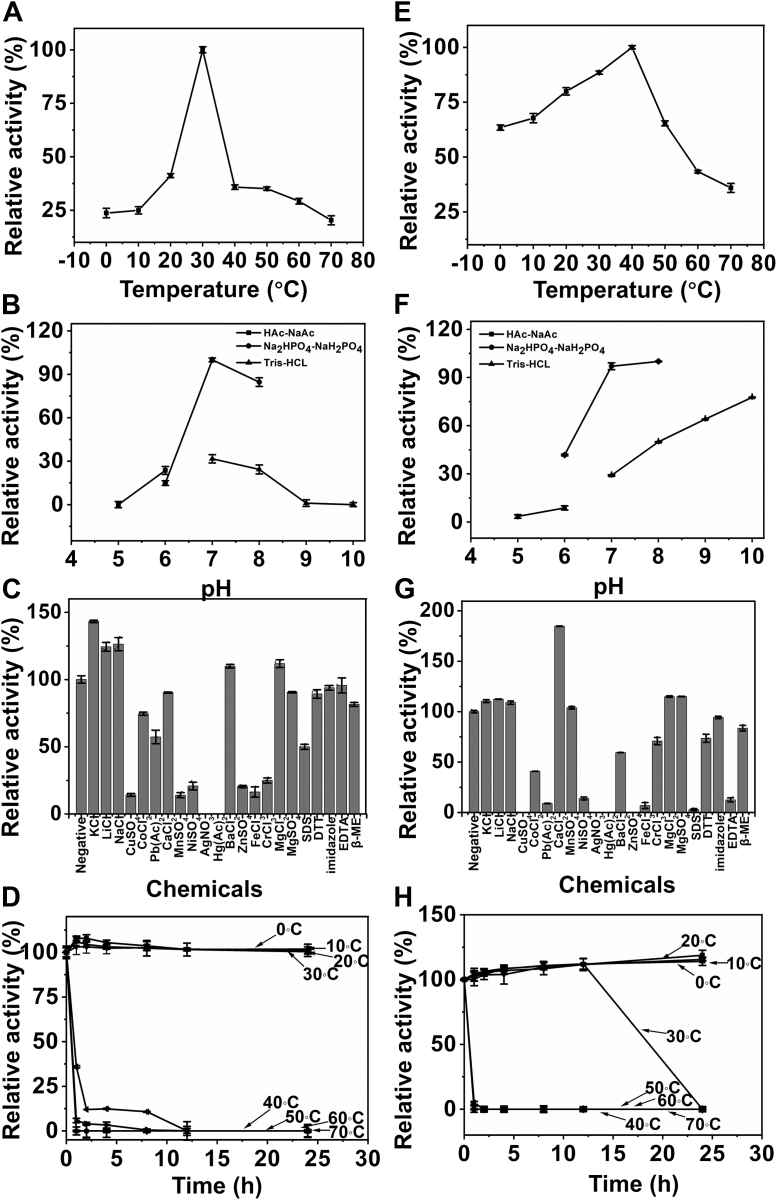
**Biochemical characteristics of recombinant IM3796 and IM1634.***A* and *E*, effects of temperature. The enzymatic activities of IM3796 (*A*) and IM1634 (*E*) were detected using CS-C as substrates in the 50 mM NaH_2_PO_4_-Na_2_HPO_4_ buffer (pH 7.0) at various temperatures (0–70 °C). The data are shown as a percentage of the activity obtained at their corresponding optimal temperatures (100%). *B* and *F*, effects of pH. The activities of IM3796 and IM1634 toward CS-C were evaluated in buffers with pH values ranging from 5 to 10 at their optimal temperatures. Data are shown as the percentage of the activity obtained in the corresponding optimal pH. *C* and *G*, effects of chemicals. The impacts of different chemicals (5 mM) towards IM3796 and IM1634 against CS-C were assessed in their respective optimal conditions. Data are shown as the percentage of the activity that acquired in the buffer without these tested chemicals. *D* and *H*, Thermostability of IM3796 and IM1634. The enzymes were incubated at different temperatures (0–70 °C) for different time interval (0–24 h), and the residual activity against CS-C was measured in the optimal conditions of each enzyme. Data are displayed as the activities relative to that of an untreated enzyme. The error bars mean of triplicate ± *SD* values.

The optimal reaction conditions of both enzymes were analyzed by using CS-C as a substrate. The temperature profiles showed that when CS-C was used as the substrate, IM3796 and IM1634 showed the highest catalytic rates at 30 °C and 40 °C, respectively, and IM1634 exhibited much wider temperature adaptability than that of IM3796 (Fig. 4, *A* and *E*). The reaction rates of both IM3796 and IM1634 against CS-C exhibited a high sensitivity toward different buffers even at the same pH, and the optimal pH values of IM3796 and IM1634 were 7.0 and 8.0 in 50 mM NaH_2_PO_4_-Na_2_HPO_4_ buffer, respectively (Fig. 4, *B* and *F*). The activity of IM3796 was significantly modestly stimulated by the basic alkali metal ions K^+^, Li^+^, and Na^+^ at a final concentration of 5 mM but almost unaffected by the divalent metal ions Mg^2+^, Ba^2+^, and Ca^2+^ as well as the chelating agent EDTA (Fig. 4*C*). In contrast, the activity of IM1634 was strongly promoted by Ca^2+^ and correspondingly inhibited by EDTA, indicating that Ca^2+^ plays a critical role in the catalytic process of this enzyme. In addition, the activities of both enzymes were strongly inhibited by Ag^+^, Co^+^, Cu^2+^, Ni^2+^, Hg^2+^, Pb^2+^, Zn^2+^, Fe^3+^, Cr^3+^, and SDS (Fig. 4, *C* and *G*). Thermostability studies revealed that IM3796 and IM1634 were quite stable and could retain 90% activity for at least 12 h at temperatures between 0 and 30 °C (Fig. 4, *D* and *H*). Notably, the activities of both enzymes quickly dropped when the temperature was increased to 40 °C, and thus, the subsequent reactions for both enzymes were carried out at 30 °C, although the optimal temperature of IM1634 was determined to be 40 °C.

Under their corresponding optimal conditions, the specific activities of IM3796 and IM1634 against HA, DS, and various CSs were measured as described under “Experimental procedures”. The specific activities of IM3796 toward CS-A, CS-C, and CS-E were 52.63, 74.37, and 14.87 mU/mg, respectively, and the enzyme activities for HA and DS were less than 1.00 mU/mg, whereas the specific activities of IM1634 against the corresponding substrates were 60.52, 40.59, 37.88, 34.80, and 41.66 U/mg, respectively. Obviously, the enzymatic activity of IM1634 was almost a thousand times higher than that of IM3796 (Table 2).

**Table 2:** Specific activities of IM3796, IM1634 and their variants against different substrates

Substrates	IM3796	IM1634	IM1634-T109	IM3796-A109
	milliunits/mg	units/mg	milliunits/mg	milliunits/mg
CS-A	52.63 ± 0.01	60.52 ± 0.05	92.28 ± 0.02	148.74 ± 0.01
CS-C	74.37 ± 0.01	40.59 ± 0.01	137.59 ± 0.04	63.31 ± 0.01
CS-E	14.87 ± 0.01	37.88 ± 0.04	48.61 ± 0.01	42.71 ± 0.01
HA	<1.00	34.80 ± 0.01	<1.00	105.43 ± 0.01
DS	<1.00	41.66 ± 0.04	18.95 ± 0.01	86.19 ± 0.01

### Substrate-degrading patterns of IM3796 and IM1634

**Figure 5:**
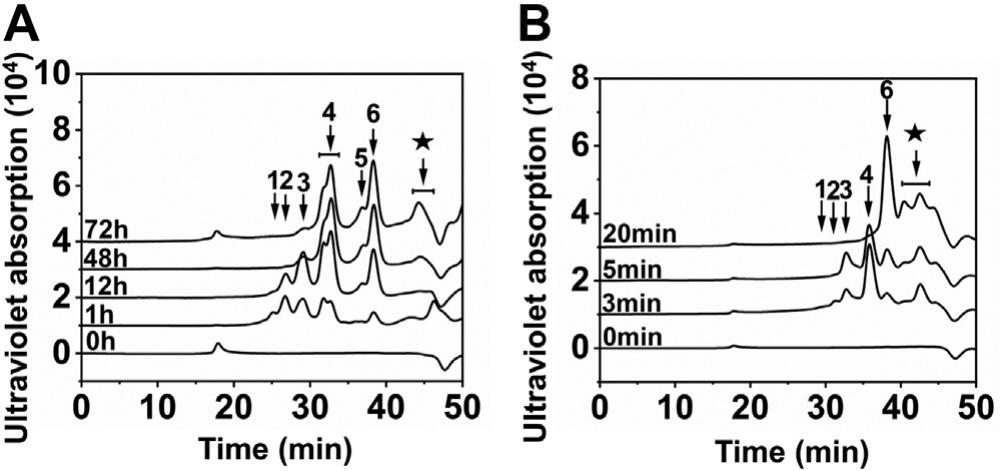
**Time-course experiments of digestion of CS-C by IM3796 and IM1634.** One hundred microliter of CS-C (1 mg/ml) or CS-A (1 mg/ml) was depolymerized by 100 μl IM3796 (10 μM) (*A*) and 20 μl IM1634 (9 μM) (*B*), and a 30 μl aliquot was taken out at different time interval and analyzed by gel filtration chromatography as described under “Experimental procedures”. The elution positions of the following CS standard oligosaccharides are indicated by *arrows*: 1, unsaturated CS decasaccharides; 2, unsaturated CS octasaccharides; 3, unsaturated CS hexasaccharides; 4 unsaturated CS tetrasaccharides; 5, unsaturated CS disulfated disaccharides; 6, unsaturated CS monosulfated disaccharides. The peaks marked with a pentagram are salt.

**Figure 6:**
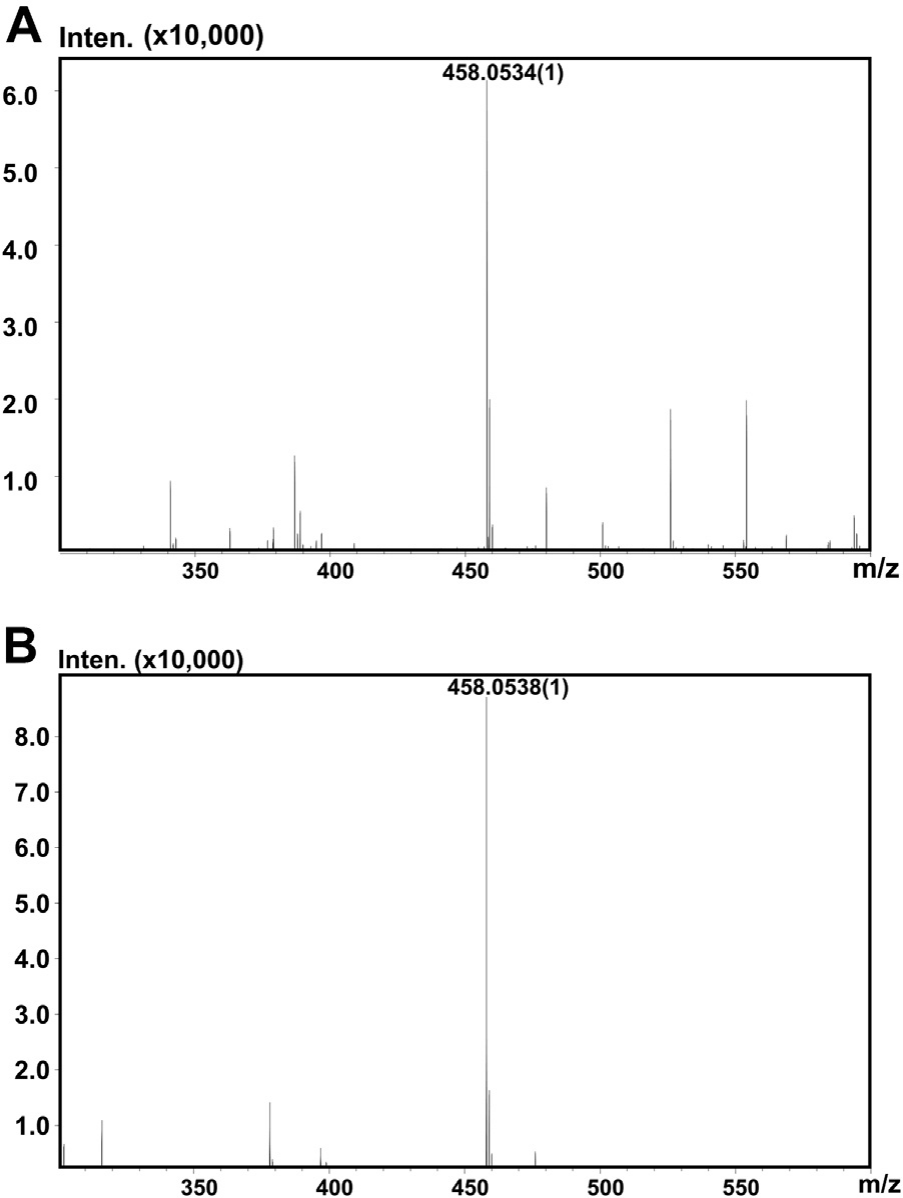
**Time-of-flight(TOF)mass spectra analysis of the monosulfated disaccharide products of IM3796and IM1634towardCS-C.** The monosulfated disaccharides from the digestion of CS-C by IM3796 (*A*) and IM1634 (*B*) were individually analyzed by ESI-MS on an IT-TOF hybrid mass spectrometer in negative ion mode.

**Figure 7:**
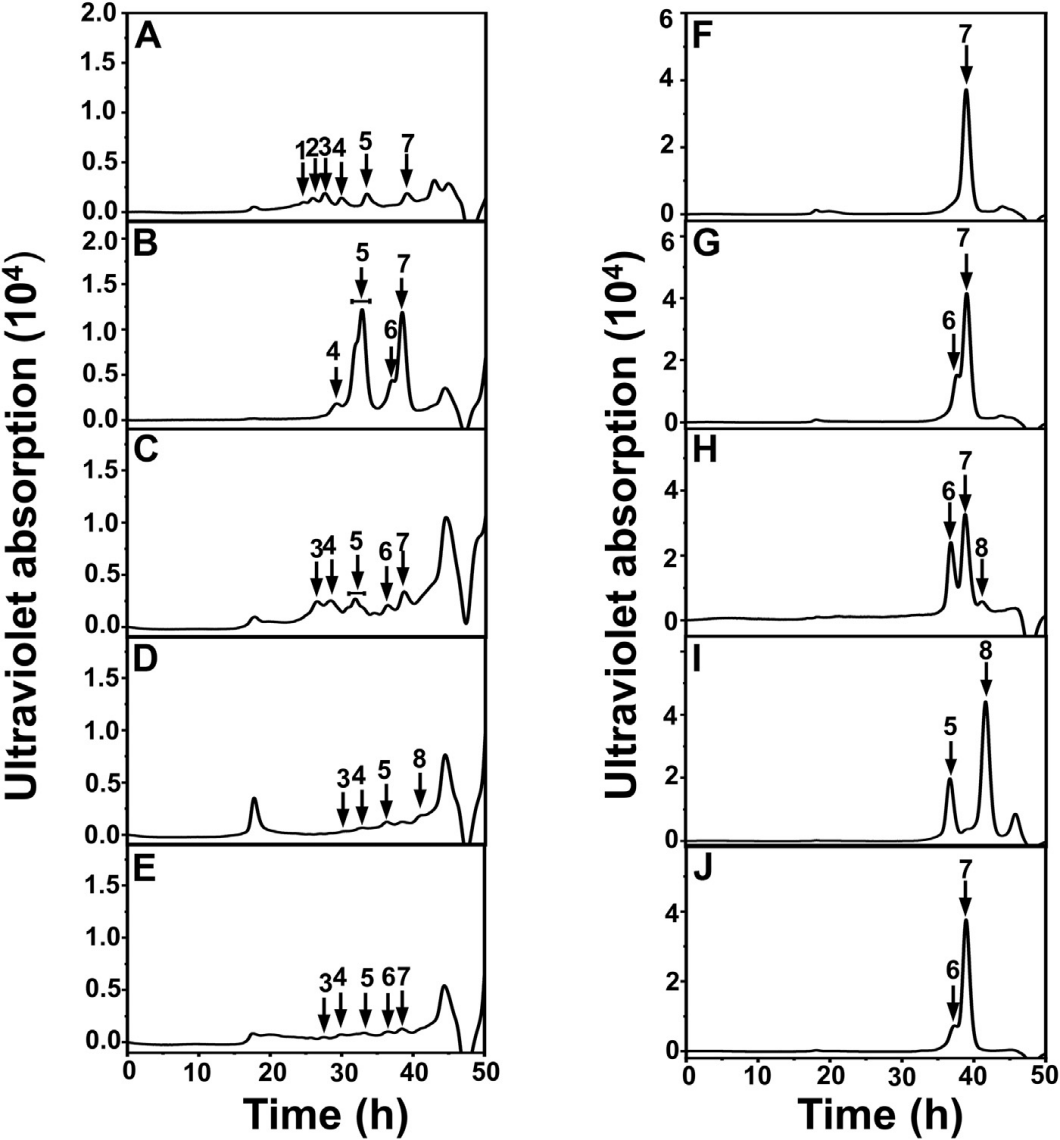
**The substrate specificity of IM3796 and IM1634 toward HA and CS/DS.** Ten micrograms of CS-A (*A* and *F*), CS-C (*B* and *G*), CS-E (*C* and *H*), HA (*D* and *I*), or DS (*E* and *J*) was digested by 10 μl IM3796 (10 μM) with the addition 2 μl fresh enzyme every 24 h up to 72 h (*A*–*E*), or by 2 μl IM1634 (9 μM) for 12 h (*F*–*J*). The products were detected by gel filtration chromatography as described under “Experimental procedures.” The elution positions of the following standard oligosaccharides are indicated by *arrows*: 1, unsaturated dodecasaccharides; 2, unsaturated decasaccharides; 3, unsaturated octasaccharides; 4, unsaturated hexasaccharides; 5, unsaturated tetrasaccharides; 6, unsaturated disulfated disaccharides; 7, unsaturated monosulfated disaccharides; 8, unsaturated nonsulfated disaccharides.

To determine the substrate-degrading pattern of IM3796 and IM1634, CS-C (1 mg/ml) and CS-A (1 mg/ml) were treated with IM3796 or IM1634 for a series of appropriate time intervals, and the resultants were analyzed by gel filtration chromatography. The results showed that both IM3796 and IM1634 initially generated large oligosaccharides and then small oligomers with increasing absorbance at 232 nm, indicating that they were both endolytic CS/DS lyases (Fig. 5). Furthermore, the main disaccharide products of CS-C digested by IM3796 and IM1634 were collected and analyzed by ESI-MS spectrometry in negative ion mode. The detected major signals emerged at *m/z* 458.0534 and 458.0538 (Fig. 6), which are consistent with the theoretical *m/z* of unsaturated monosulfated Δ^4,5^HexUA-GalNAc(6/4S) produced from the digestion of CS-C by CS/DS lyases *via* a *β*-elimination mechanism. This result further confirmed that both IM3796 and IM1634 were lyases. Moreover, the products of HA, DS, CS-A, CS-C, and CS-E exhaustively digested by IM3796 and IM1634 were analyzed by gel filtration chromatography. The results showed that IM3796 could well digest CS-C containing 76.25% 6-*O*-sulfated GalNAc into tetra- and disaccharides but only partially degraded CS-A, mainly containing 4-*O*-sulfated GalNAc, and CS-E, rich in 4,6-*O*-sulfated GalNAc, and slightly degraded HA and DS (Fig. 7, *A*–*E*), indicating that this enzyme tends to act on CS rich in 6-*O*-sulfated GalNAc residues. In contrast, IM1634 could completely digest various CSs and DS into disaccharides and HA into tetra- and disaccharides (Fig. 7, *F*–*J*). The large difference in enzymatic properties suggests that the N-terminal extra peptide of IM1634 may play an important role in regulating the substrate selectivity and product profile of the two homologous enzymes.

### Analyzing the disaccharide composition of oligosaccharides produced from the exhaustive digestion of CS-C by IM3796

To investigate whether IM3796 tended to degrade domains containing 6-*O*-sulfated GalNAc, CS-C was exhaustively digested by IM3796, and the final products were fractionated by gel filtration. The main digests, including di-, tetra-, and hexasaccharide fractions, were individually collected and treated with CSase ABC to analyze the disaccharide composition. As shown in Table 3, the proportions of the 6-*O*-sulfated GalNAc-containing Δ^4,5^HexA1-3GalNAc(6S) (ΔC) and Δ^4,5^HexA(2S)1-3GalNAc(6S) (ΔD) were significantly increased from 45.28% in hexasaccharides to 83.19% in disaccharides, while those of 4-*O*-sulfated GalNAc-containing Δ^4,5^HexA1-3GalNAc(4S) (ΔA) were decreased from 54.72% in hexasaccharides to 16.81% in disaccharides. These results clearly show that 6-*O*-sulfated ΔC and ΔD rather than 4-*O*-sulfated ΔA are enriched in the minimal product disaccharides, further suggesting that IM3796 prefers to digest domains containing the 6-*O*-sulfated but not 4-*O*-sulfated GalNAc residues in CS chains.

**Table 3:** Disaccharide compositions of oligosaccharides from the exhaustive digestion of CS-C by IM3796

Mol%^b^ Oligosaccharide	ΔO^a^	ΔC	ΔA	ΔD	ΔE
CS-C	ND^c^	56.03	23.75	20.22	ND
CS-C hexasaccharide	ND	30.49	54.72	14.79	ND
CS-C tetrasaccharide	ND	52.11	33.00	14.89	ND
CS-C disaccharide	ND	57.59	16.81	25.60	ND

a The following abbreviations represented unsaturated disaccharide units: ΔO (Δ^4,5^HexA1–3GalNAc); ΔC (Δ^4,5^HexA1–3GalNAc(6S)); ΔA (Δ^4,5^HexA1–3GalNAc(4S)); ΔD (Δ^4,5^HexA(2S)1–3GalNAc(6S)); ΔE (Δ^4,5^HexA1–3GalNAc(4S,6S)).

b The molar ratio of disaccharides was calculated based on their peak area.

c ND means not detected.

### Characterization of structures susceptible to digestion by IM3796

**Figure 8:**
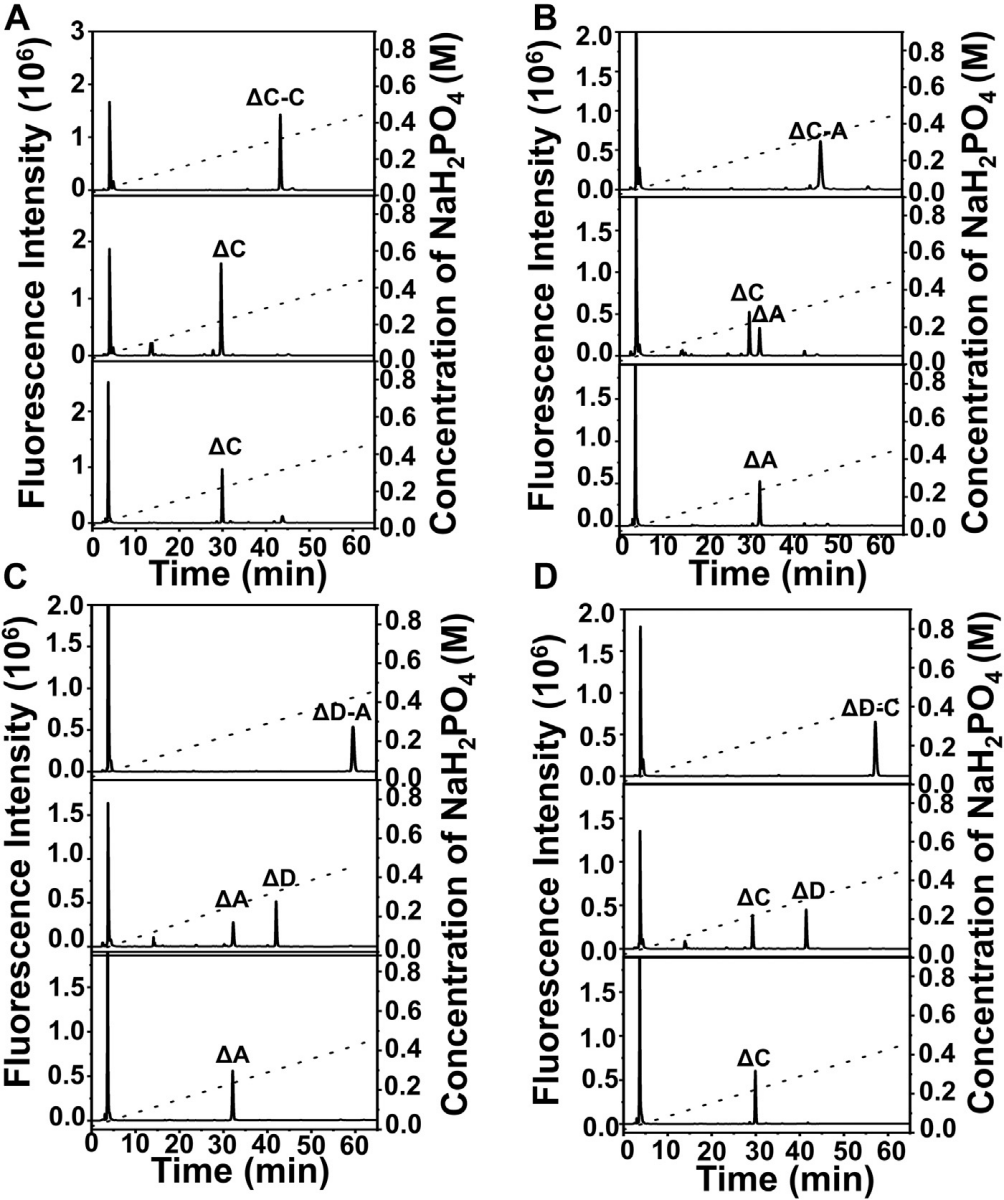
**Sequencing of tetrasaccharide products of IM3796 toward CS-C.***Top panel*, Isolated tetrasaccharide subfraction labeled with 2-AB; *middle panel*, the disaccharide composition of each tetrasaccharide subfraction; *Bottom panel*, the reducing-end disaccharide of each tetrasaccharide subfraction. All of the samples were analyzed on a YMC-Pack PA-G column using a linear gradient from 16 to 460 mM NaH_2_PO_4_ solution (shown by the *dotted line*) during a 60 min period and monitored by a fluorescence detector at excitation and emission wavelengths of 330 and 420 nm, respectively. The authentic 2-AB-derivatized unsaturated CS disaccharides were used as standards. *A*, ΔC-C; *B*, ΔC-A; *C*, ΔD-A; *D*, ΔD-C.

To characterize the susceptible structures to digestion by IM3796, the minimal size of substrates was first determined by using size-defined oligosaccharides prepared from the partial digestion of CS-C by CSase ABC that could cleave CS/DS randomly. The results showed that IM3796 could not degrade tetrasaccharides but could efficiently cleave hexasaccharides into tetrasaccharides and disaccharides with similar molar ratios, suggesting that the minimal substrate of IM3796 was hexasaccharide, although some hexasaccharides were resistant to digestion (Fig. S3). To further investigate the specific structures preferred by IM3796, the tetrasaccharide fraction from the exhaustive digestion of CS-C was subfractionated by anion-exchange HPLC on a YMC-Pack PA-G column, and four major subfractions (Fr.1–4) were detected and collected for subsequent structural characterization (Fig. S4). The sequences of tetrasaccharides in Fr.1–4 were analyzed by an enzymatic method as described previously (35, 39). The results showed that the major tetrasaccharides corresponding to Fr.1–4 in the final product of CS-C were ΔC-C (Fig. 8*A*), ΔC-A (Fig. 8*B*), ΔD-A (Fig. 8*C*), and ΔD-C (Fig. 8*D*). The structures of four tetrasaccharides were confirmed by mass spectrometry. Unsaturated disulfated ΔC-C and unsaturated disulfated ΔC-A were assigned to m/z 458.0452 or m/z 458.0472 with two charges, while unsaturated trisulfated ΔD-A and unsaturated trisulfated ΔD-C group were assigned to m/z 498.0243 or m/z 498.0247 with two charges and m/z 331.6827 or m/z 331.6804 with three charges. The MS spectra showed a consistent theoretical mass with these four tetrasaccharides for 918.1366 and 998.0934, respectively (Fig. S5). Based on the structures of the main tetrasaccharide products, the unsaturated disaccharides located at the nonreducing ends of the four tetrasaccharides clearly all contain a 6-*O*-sulfated GalNAc residue. These results suggest that IM3796 tends to act on the uronic acid residues linked to 6-*O*-sulfated GalNAc residues regardless of whether the uronic acid residues are 2-*O*-sulfated, which further confirms the substrate preference of this enzyme.

### Structural modeling and mutation analysis of IM3796 and IM1634

**Figure 9:**
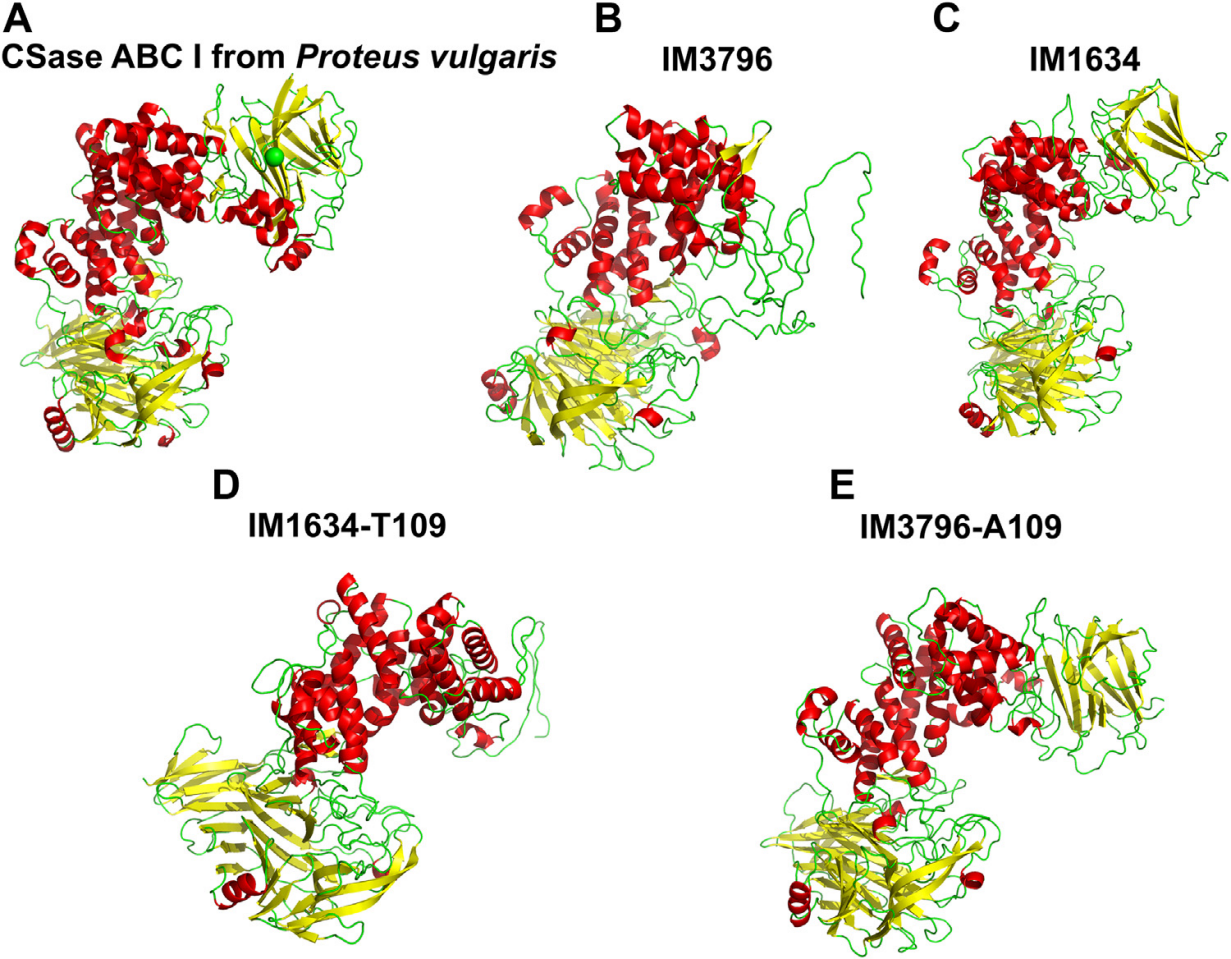
**The tertiary structure modeling of IM3796, IM1634, and their variants.***A*, the crystal structure of CSase ABC I from *Proteus vulgaris*. *B*–*E*, the tertiary structure modeling of IM3796, IM1634, IM1634-T109, and IM3796-A109 were carried out using SWISS-MODEL software online.

To preliminarily explore the structural basis of the difference in enzymatic features between IM3796 and IM1634, the three-dimensional structures of IM3796 and IM1634 were simulated with SWISS-MODEL software online using the structure of CSase ABC I from *P. vulgaris* (40) as a template (https://swissmodel.expasy.org/) (Fig. 9, *A*–*C*). The modeling structures show that both IM3796 and IM1634 contain middle (catalytic) and C-terminal domains very similar to those of the template CSase ABC I, but IM3796 is missing the N-terminal domain with a two-layered bent β-sheet sandwich that is found in IM1634 and CSase ABC I (Fig. 9, *A*–*C*). The N-terminal domain of CSase ABC I is highly similar to the carbohydrate-binding domains of many proteins, such as xylanases, glucanases, and some lectins, suggesting that this domain may be involved in the binding of GAG substrates (40). However, the real role of this domain in the catalytic mechanism of CSase ABC I-like enzymes remains to be investigated further.

**Figure 10:**
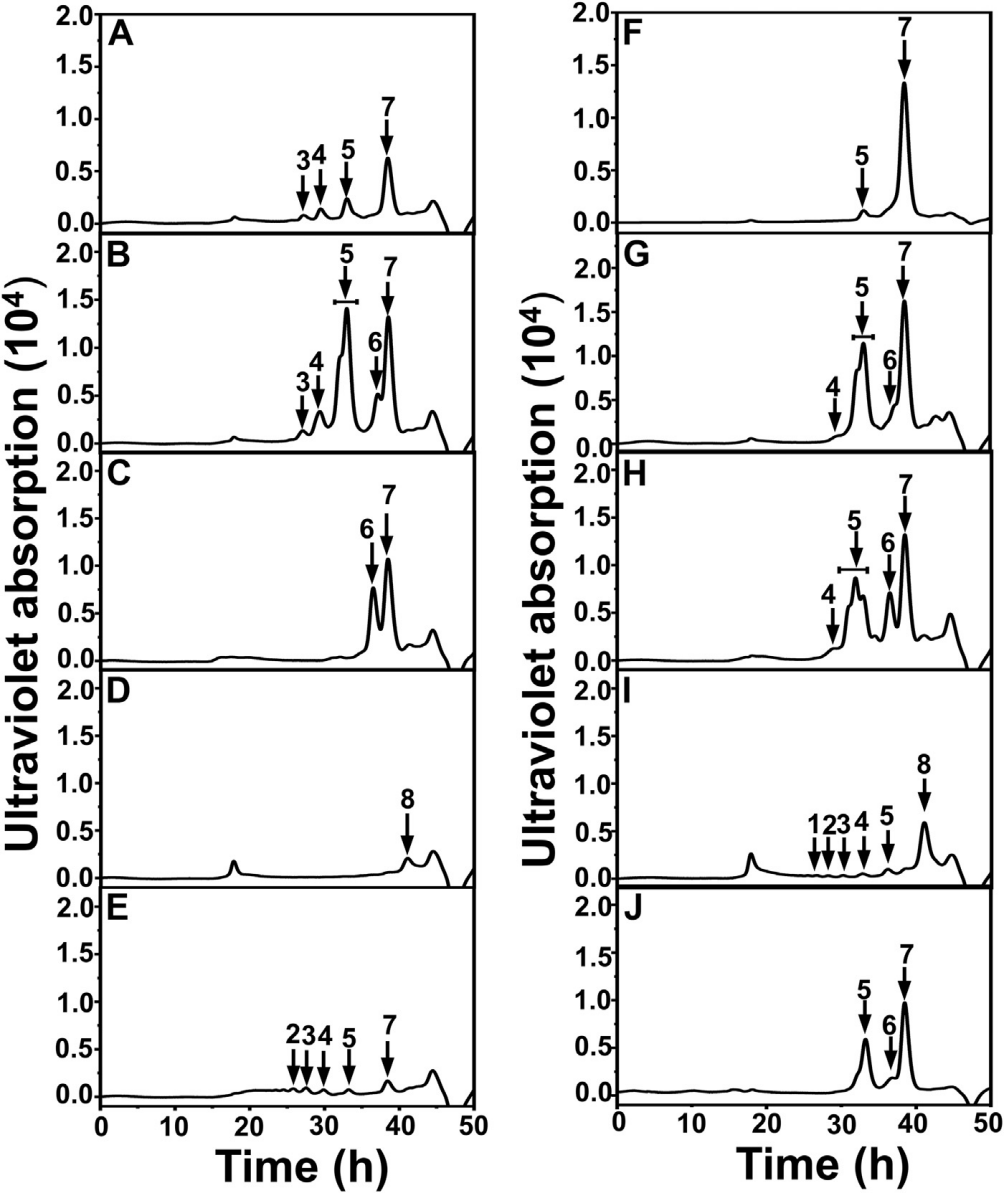
**The substrate specificity of IM1634-T109 and IM3796-A109 toward HA and CS/DS.** Ten micrograms of CS-A (*A* and *F*), CS-C (*B* and *G*), CS-E (*C* and *H*), HA (*D* and *I*), or DS (*E* and *J*) was digested by 5 μl IM1634-T109 (10 μM) (*A*–*E*) or IM3796-A109 (9 μM) (*F*–*J*) with the addition 2 μl corresponding fresh enzyme every 24 h up to 48 h. The final products were detected by gel filtration chromatography as described under “Experimental procedures.” The elution positions of the following standard oligosaccharides are indicated by *arrows*: 1, unsaturated dodecasaccharides; 2, unsaturated decasaccharides; 3, unsaturated octasaccharides; 4, unsaturated hexasaccharides; 5, unsaturated tetrasaccharides; 6, unsaturated disulfated disaccharides; 7, unsaturated monosulfated disaccharides; 8, unsaturated nonsulfated disaccharides.

To investigate the possible function of the N-terminal domain composed of two β-sheets, the additional N-terminal sequence (Met^1^-His^109^) present in IM1634 but not IM3796 was deleted or added to the N-terminus of IM3796 to prepare the truncated variant IM1634-T109 and the grafted variant IM3796-A109, respectively (Fig. S1). Structure modeling showed that the N-terminal domain was absent in IM1634-T109, similar to IM3796, but present in IM3796-A109, similar to IM1634 (Fig. 9, *D* and *E*). Furthermore, the enzymatic analysis showed that the activities of the truncated IM1634-T109 against various substrates decreased by almost a thousand times compared with that of IM1634 (Table 2), and the enzyme could no longer completely degrade CS/DS into disaccharides, as observed with IM1634, and very weakly acted on CS-A, DS, and HA, similar to IM3796 (Figs. 6 and 10). In addition, IM1634-T109 could cleave CS-C hexasaccharide into tetra- and disaccharide but failed to digest CS-C tetrasaccharide (Fig. S6), just like the case of IM3796 (Fig. S3). Conversely, the grafted variant IM3796-A109 with the N-terminal sequence (Met^1^-His^109^) shows remarkably higher activities than that of its parental IM3796 toward various substrates and can even efficiently degrade CS-A, CS-E, and DS into tetra-/disaccharides (Table 2 and Fig. 10, *F*–*J*). These results demonstrate that the N-terminal domain plays an important role in regulating the substrate-binding and substrate-degrading capacity of these enzymes.

## Discussion

CSase ABCs are important tools in structural and functional studies of CS/DS (27, 28, 29, 30, 31, 32) and promising therapeutic agents for axon regeneration (41). Although a few CSase ABCs have been identified from *P. vulgaris*, *B. stercoris*, *F. heparium*, *Photobacterium* sp., and *Vibrio* sp., most of the research was focused on preliminary studies of basic characteristics, such as the optimal reaction conditions and specific activities against different polysaccharide substrates; thus, detailed investigation of their substrate-degrading pattern as well as related regulation mechanism is lacking (33, 34, 35), which greatly limits the ability to apply the corresponding enzymes and engineer modifications. In the present study, two CSase ABCs, IM3796 and IM1634, were identified from the intestinal metagenome and comparatively studied through detailed biochemical and structural analyses. Both IM3796 and IM1634 share very low sequence similarity (≤31%) with all identified GAG lyases, and each enzyme shows unique enzymatic characteristics different from the identified CSase ABCs, suggesting the novelty and potential application of IM3796 and IM1634. In particular, these two enzymes are highly homologous in sequence similarity (query coverage: 88.00%, percent identity: 90.10%), but they show very different enzymatic properties, such as substrate selection and product generation. Furthermore, sequence alignment combined with structural simulation and mutation assays demonstrated that the absence of the N-terminal domain was a key reason why the enzymatic properties of IM3796 and IM1634 are different, indicating that the N-terminal domain of CSase ABCs functions as a regulator of substrate binding and degradation, as speculated previously (40).

According to the substrate-degrading pattern, CSase ABCs can be classified into endolytic and exolytic types. The most commonly used commercial “CSase ABC” is actually a mixture of endolytic CSase ABC I and exolytic CSase ABC II extracted from *P. vulgaris* (42). Phylogenetic and biochemical analyses showed that both IM3796 and IM1634 belong to the type of endolytic CSase ABC I, but their substrate preferences are obviously different. In most cases, CSase ABCs have a broad substrate spectrum and can efficiently digest various CSs, DS, and HA with varying degrees of activity; however, there are exceptions, such as two exolytic CSase ABCs from *B. stercoris* HJ-15 and *Flavobacterium heparinum*, which were reported to fail to degrade HA (34, 36). Similar to most of the identified CSase ABCs, IM1634 exhibits high activity toward various CSs, DS, and HA. However, IM3796 is very unique compared with other CSase ABCs, although it is classified as a CSase ABC I type. Analysis of the specific activities and final products of IM3796 against various GAG substrates showed that compared to other CSs, DS, and HA, this enzyme showed a much greater preference to degrade 6-*O*-sulfated GalNAc-enriched CS-C (Table 2 and Fig. 7), which is very similar to the CSase C isolated from extracts of *F. heparinum* induced by CS/DS (25). Notably, “CSase C” has not been recombinantly expressed because its gene is unclear, and thus, identifying IM3796 will provide a new and reliable option for utilizing “CSase C” in some studies and applications related to 6-*O*-sulfated CS.

In terms of the final products, exolytic CSase ABCs (type II) can completely digest CS/DS as well as HA into disaccharides by sequentially releasing unsaturated disaccharides from the reducing or nonreducing ends of these substrates (34, 36, 40), while most endolytic CSase ABCs (type I) randomly act on CS/DS to generate unsaturated tetrasaccharides and disaccharides (23, 40). IM3796 could digest its preferred CS-C into tetra- and disaccharides as the main final products, which is common for endolytic CSase ABCs, such as CSase ABC I from *P. vulgaris* (ATCC6896) (33) and enCSase from *Photobacterium* sp (35). However, IM3796 produced a series of oligosaccharide products with varying sizes when acting on CS-A, CS-E, DS and HA due to its high preference for 6-*O*-sulfated CS (Fig. 6). Unlike most endolytic CSase ABCs, IM1634 could completely degrade CS/DS into disaccharides, which is equivalent to the combined effect of CSase ABC I and II in the commercial “CSase ABC” from *P. vulgaris*. A CSase ABC Vpa_0049 from the marine bacterium *Vibrio* sp. QY108 was reported to completely digest various CSs and DS into disaccharides, but interestingly, characteristic disulfated disaccharide ΔD was not detected in the resultants of CS-C and CS-D digested by this enzyme (37). In contrast, the high equality to the commercial “CSase ABC” in substrate spectrum and product profile suggests that IM1634 will be a very useful tool in the structural and functional studies of CS/DS.

Compared with IM1634, IM3796 lacks 109 amino acids at the N-terminus, which leads to a loss of the two-layered bent β-sheet domain in the modeling structure (Fig. 9*B*). It has been speculated that the N-terminal domain of CSase ABC I from *P. vulgaris* assists in binding to the long GAG chain based on its structural similarity to the carbohydrate-binding domains of other proteins (40). This study provides direct evidence for the role of the N-terminal domain in facilitating substrate binding by comparing the enzymatic properties of IM3796 and IM1634 as well as their respective grafted and truncated variants. As we mentioned earlier, much lower enzymatic activity was observed for IM3796 without this domain than for IM1634. In contrast to IM1634, IM3796 cannot efficiently degrade various CS/DS to disaccharides, even though IM3796 and IM1634 are highly homologous in sequence and IM3796 tends to degrade CS-C only into tetra- and disaccharides. When the N-terminus (Met^1^–His^109^) of IM1634 was grafted to IM3796, the resulting variant IM3796-A109 became more efficient at degrading various CS/DS and HA compared to its parental IM3796. However, the activity of IM3796-A109 is still quite low compared to that of IM1634 (Table 2), which may be due to that the linker between the N-terminal domain of IM1634 and IM3796 requires to be optimized to ensure the correct steric arrangement of the structural domains for high enzymatic activity. In addition, the rest sequences of IM3796 and IM1634 are highly homologous but not completely identical without considering their N-terminal domains, which can be another reason why the activity of the grafted enzyme IM3796-A109 is not comparable to that of IM1634. On the other hand, the truncation of the N-terminus (Met^1^–His^109^) from IM1634 caused the variant IM1634-T109 dramatically lost enzymatic activity toward various CS/DS and HA, could not completely degrade CS/DS into disaccharides, and exhibited a significant preference for CS-C and CS-E. Taken together, these results demonstrate that the N-terminal domain is critical for the substrate selectivity and product generation of enzymes, which occurs because this domain facilitates the binding of substrates, particularly low- and nonsulfated substrates such as CS-A and HA. The preference of IM3796 for CS-C occurs because the structure between the catalytic site of the enzyme and GalNAc residue is easily adapted with 6-*O*-sulfation rather than others because 6-*O*-sulfated GalNAc-containing disaccharides ΔC and ΔD, as well as ΔC-/ΔD-containing tetrasaccharides, were mainly detected in the final digest of CS-C. Furthermore, deleting the N-terminal domain resulted in decreased activity but an increased substrate preference for the truncated variant IM1634-T109. Notably, both IM3796 and IM1634-T109 failed to digest tetrasaccharide, indicating that the N-terminal domain is also essential for the binding of short substrate. Taken together, the N-terminal domain plays a key role in regulating the enzymatic activity and substrate selectivity of CSase ABC I, which can be an important target when engineering these enzymes in the future.

In conclusion, the detailed comparative study of two homologs, IM3796 and IM1634, provides two new enzymes with unique enzymatic properties for CS/DS-related studies and applications and, more importantly, reveals the key role of the N-terminal domain in regulating the substrate binding and degradation of these enzymes, which will be a helpful reference for future structure‒function studies and for engineering of CSase ABC.

## Experimental procedures

### Materials

The putative enzyme gene *IM3796* was cloned from the metagenome DNA extracted from the feces of the KM mice drinking water supplemented with 1% (w/v) CS-A for 3 months. The related strains and plasmids used in this study are listed in Table 1. PrimeSTAR HS DNA polymerase was purchased from Takara, Inc. LightNing DNA Assembly Mix Plus was purchased from Yugong Biolabs Co, Ltd. *E. coli* competent cells, FastPure Gel DNA Extraction Mini Kit, FastPure Plasmid Mini Kit, and ClonExpress II One Step Cloning Kit were purchased from Vazyme Biotech Co, Ltd. The bicinchoninic acid (BCA) protein test kit was purchased from Cowin Bio Co, Ltd. Alginate, HA from *Streptococcus equi* (15–30 kDa), CS-A from bovine cartilage (43), 2-aminobenzamide (2-AB), and cyanoborohydride (NaBH_3_CN) were obtained from Sigma Aldich. CSase ABC (EC 4.2.2.20), CS-C from shark cartilage, CS-E from *Dosidicus gigas* cartilage (44), and DS and HS from porcine intestinal mucosa were obtained from GlycoSciTech Co, Ltd. Hep from porcine intestinal mucosa was provided by Tiandong Pharma (43). Standard GAG unsaturated disaccharides were purchased from Iduron. The rest of the commercial chemicals and reagents were of the highest quality available. CS-C tetra-and hexasaccharides were prepared by the partial degradation of CS-C with CSase ABC followed by gel filtration chromatography on a Superdex Peptide 10/300 GL column as described previously (35). The KM mice weighing between 30.0 and 35.0 g were obtained from Qingdao Daren Fucheng Animal Husbandry Co and raised in a comfortable environment, fed standard food, and allowed to drink freely. All animal experiments were approved by the Animal Care and Use Committee of Shandong University.

### Prediction of IM3796 and IM1634 sequences in the intestinal metagenome

The fecal samples were obtained from KM mice drinking 1% CS-A (w/v) for 3 months. The total genomic DNA was extracted, sonicated, and used to construct a Paired-end library with an average size of about 400 bp. The Illumina NovaSeq/Hiseq Xten (Illumina Inc) was used for sequencing. The quality of raw data was checked by fastp (https://github.com/OpenGene/fastp) and then high-quality reads were acquired and assembled by Megahit (https://github.com/voutcn/megahit) (45). The assembled contigs were predicted by MetaGene (http://metagene.cb.k.u-tokyo.ac.jp/) (46) and then the predicted proteins IM3796 and IM1634 were annotated by the Carbohydrate Active enZYme (CAZy) (http://www.cazy.org/) (38, 47).

### Genes and proteins sequence analysis of IM3796 and IM1634

Primer Premier version 5.0 (PREMIER Biosoft International) and the Promoter 2.0 Prediction Server were used to find promoter motifs in the 5′-flanking DNA region upstream of the ORF. The GC content (G + C%) of the ORF was calculated using Bio-Edit version 7.2.5. The theoretical molecular weight and pI were both calculated through the ExPASy server. The signal peptide was predicted using the SignalP 5.0 server. Sequence similarity analysis was performed by the online BLAST algorithm through the National Center for Biotechnology Information program. The phylogenetic analysis was performed with MEGA 7.0.26 tool by Neighbor-Joining methodology and 1000 bootstrap replications.

### Cloning, expression, and purification of recombinant enzymes

The putative genes *IM3796* and *IM1634* from the metagenomic DNA of mouse feces were amplified using the sequence-specific primer pairs (as listed in Table 1) and high-fidelity PrimeSTAR HS DNA polymerases (Takara Inc). The amplicons were cloned into the pET-30a vector (Novagen) using one-step cloning using ClonExpress II One Step Cloning Kit, followed by DNA sequencing, and then transformed into *E. coli* BL21 (DE3) cells (Novagen) for the overproduction of the recombinant proteins IM3796 and IM1634, respectively (48).

*E. coli* cells harboring the recombinant plasmids pET30a-*IM3796* or pET30a-*IM1634* were cultured in LuriaBertani (LB) medium containing 50 μg/ml kanamycin. The cells were grown at 37 °C and 200 rpm until the cell density reached an A600 of 0.8 to 1.0. For expression of the target proteins, the broth was supplemented with the inducer isopropyl-1-thio-β-D-galactoside (IPTG) at a final concentration of 0.05 mM to initiate the expression of targeting proteins. After being cultured at 16 °C for another 24 h, the cells were collected by centrifugation at 8000 rpm for 10 min, washed twice, resuspended with ice-cold buffer A (50 mM Tris-HCl, 150 mM NaCl, pH 8.0), and disrupted by sonication (72 repetitions, 4 s) in an ice-cold environment. The supernatant containing the soluble putative enzyme was collected by centrifugation at 12,000 rpm for 30 min in 4 °C, loaded on a column packed with nickel-Sepharose six Fast Flow (GE Healthcare) equilibrated with buffer A supplementing 5 mM imidazole to remove impurities, and then eluted with a linear gradient of buffer A containing 50 to 250 mM imidazole to acquire the target protein. The molecular mass and purity of the purified enzyme were determined using 13.2% (w/v) sodium dodecyl sulfate-polyacrylamide gel electrophoresis (SDS-PAGE) (49). The imidazole in the elution fractions was removed using ultrafiltration (Amicon Ultra 0.5-ml 10 K unit, Millipore), and the concentration of the recombinant protein was measured by BCA protein test kit.

### The specificity of IM3796 and IM1634 toward various polysaccharide substrates

To determine the substrate specificity of these two enzymes, 150 μl of various polysaccharides (1 mg/ml), including HA, CS-A, CS-C, CS-E, DS, alginate, Hep and HS, were treated with 15 μl of 10 μM IM3796 for 2 h at 30 °C or 2 μl of 9 μM IM1634 for 10 min at 30 °C in the 50 mM NaH_2_PO_4_-Na_2_HPO_4_ buffer (pH 7.0), and the reactions were terminated by boiling. After centrifugation, the absorbance of the supernatants at 232 nm was measured to determine the ability of each enzyme to degrade various substrates (23).

### Biochemical characterization of IM3796 and IM1634

The optimal pH of IM3796 and IM1634 were determined by using CS-C as substrate and measuring their reaction rates at different pH values, including 50 mM NaAc-HAc buffer (pH 5.0–6.0), 50 mM NaH_2_PO_4_-Na_2_HPO_4_ buffer (pH 6.0–8.0), and 50 mM Tris-HCl buffer (pH 7.0–10.0), at 30 °C for 2 h or 10 min, respectively. Their optimal temperatures were individually determined in their optimal buffer at temperatures ranging from 0 to 70 °C. The effects of chemicals (5 mM) on the IM3796 and IM1634 were examined by assessing the reaction rates under their optimal conditions. To test the thermostability of IM3796 and IM1634, enzymes were preincubated for 0 to 24 h at various temperatures range from 0 to 70 °C, and their residual activities were measured separately under their optimal conditions. All of the reactions were carried out in triplicate. The enzyme activities were determined by measuring the absorbance at 232 nm (23).

To determine the specific activities of IM3796 and IM1634 towards HA, CS-A, CS-C, CS-E, and DS, 150 μg of each substrate was digested with purified 45 μl IM3796 (10 μM) or 2 μl IM1634 (9 μM) under the optimal conditions of the corresponding enzyme. The enzymatic reactions were performed from 30 s to 5 min and terminated by boiling, and the enzyme activities were determined by measuring the absorbance at 232 nm. One unit of the enzyme was defined as the amount of enzyme that generated 1 μmol of unsaturated carbon bonds per minute (23).

### Substrate-degrading patterns of IM3796 and IM1634

Either endolytic or exolytic activity of IM3796 and IM1634 was first investigated by using a time-course assay as described previously (23). Briefly, CS-C (1 mg/ml) and CS-A (1 mg/ml) were, respectively, depolymerized by IM3796 and IM1634 at their optimal conditions, and aliquots (30 μl) of the reactants were taken out and immediately terminated at different time points for gel filtration analysis using a Superdex Peptide 10/300 GL column (GE Healthcare) monitored at 232 nm by a UV detector. To determine the oligosaccharide profiles of the products of various polysaccharide substrates exhaustively digested by each enzyme, HA, CS-A, CS-C, CS-E, and DS (10 μg) were individually digested by 10 μl IM3796 (10 μM) at 30 °C for 72 h in the 50 mM NaH_2_PO_4_-Na_2_HPO_4_ buffer (pH 7.0) with the addition fresh enzyme (2 μl) every 24 h or 2 μl IM1634 (9 μM) at 30 °C for 12 h in the 50 mM NaH_2_PO_4_-Na_2_HPO_4_ buffer (pH 8.0). The corresponding reactants were analyzed by gel filtration chromatography as described earlier. To determine the minimum substrate of IM3796, CS-C tetrasaccharides and hexasaccharides were digested with IM3796 under optimal conditions. The final products were analyzed by gel filtration chromatography too.

The monosulfated disaccharides isolated from the digest of CS-C treated with IM3796 or IM1634 were identified by ESI-MS on an IT-TOF (LCMS-IT-TOF) hybrid mass spectrometer (Shimadzu). The ESI-MS analysis was performed in negative ion mode with the following settings: 3.6 kV source voltage, 1.5 l/min nebulizer nitrogen gas flow rate, 200 °C heat block and curved desolvation line temperature, and 1.8 kV detector voltage. The range for the mass acquisition was set at 200 to 1000.

### Disaccharide composition assay

Disaccharide compositions of various CS/DS were analyzed by high HPLC as described previously (27). Briefly, CS/DS polysaccharides or oligosaccharides were individually digested by CSase ABC followed by 2-AB labeling and analyzed by anion exchange HPLC on a YMC Pack PA-G column (YMC-Pack) eluted with a linear gradient from 16 mM to 460 mM NaH_2_PO_4_ over 60 min at a flow rate of 1.0 ml/min at room temperature using a fluorescence detector with excitation and emission wavelengths of 330 and 420 nm, respectively. Identification and quantification of the resulting disaccharides were achieved by comparison with the elution positions of CS-derived authentic unsaturated disaccharides.

### Sequencing of main tetrasaccharides in the final products of CS-C digested by IM3796

The tetrasaccharide fractions from the final digest of CS-C by IM3796 were subfractionated by anion-exchange HPLC on a YMC Pack PA-G column using a linear gradient from 16 mM to 1 M NaH_2_PO_4_ during a 60 min period at a flow rate of 1.0 ml/min. The eluates were monitored by a UV detector at 232 nm. The major fractions were collected and desalted with gel filtration chromatography on a Superdex Peptide 10/300 GL column. Commercial CSase ABC (EC 4.2.2.20) and HCDLase (39) were used to sequence the purified tetrasaccharide subfractions as previously reported (35, 50). Briefly, to identify the disaccharide composition, each tetrasaccharides fraction (5 pmol) was digested with CSase ABC and labeled with 2-AB. Meanwhile, the corresponding tetrasaccharides were first labeled with 2-AB and then degraded by HCDLase to determine the disaccharide at the reducing ends. All samples were individually analyzed through anion-exchange HPLC on a YMC Pack PA-G column as described earlier. The tetrasaccharides were further identified by ESI-MS on an IT-TOF hybrid mass spectrometer as described above. The range for the mass acquisition was set at 200 to 1000.

### Structural modeling and mutation analysis of IM3796 and IM1634

To explore the probable catalytic mechanisms of IM3796 and IM1634, homology modeling was first performed to predict the three-dimensional structure of IM3796 and IM1634 using the structure (PDB ID: 1HN0) of CSase ABC I from *P. vulgaris* as a template through SWISS-MODEL software online (https://swissmodel.expasy.org/) (40, 51). To investigate the potential function of the extra N-terminal sequence (Met^1^–His^109^) of IM1634, a truncated variant of IM1634, IM1634-T109 without the N-terminal Met^1^–His^109^ was constructed by using PrimeSTAR HS DNA polymerase, and a grafting variant of IM3796-A109 added the N-terminal Met^1^-His^109^ of IM1634 was constructed by using LightNing DNA Assembly Mix Plus (Yugong Biolabs). Corresponding primer pairs are listed in Table 1. All PCR products were ligated to the expression vector pET-30a (+), and the expression vector was transformed into *E. coli* BL21 (DE3) for expressing corresponding variants. The homology modeling of IM1634-T109 and IM3796-A109 was performed using SWISS-MODEL software online. The substrate-degrading patterns and enzyme activities of variants were measured as described above. The activity of IM1634 and IM1634-T109 against CS-C tetra- or hexasaccharide was also assayed as described for IM3796 above.

## Data availability

All data supporting the findings of this study are available within the paper (and its supporting information files). All relevant data generated during this study or analyzed in this published article are available from the corresponding author on reasonable request. Source data are provided with this paper.

## Supporting information

This article contains supporting information (43, 44, 52, 53).

## Conflict of interest

The authors declare that they have no conflicts of interest with the contents of this article.
