# Supplementary material for: Enzymatic comparison of two homologous enzymes reveals N-terminal domain of chondroitinase ABC I regulates substrate selection and product generation
Source: J Biol Chem. 2023 Apr 7;299(5):104692. doi: 10.1016/j.jbc.2023.104692 (PMC10197112; doi:10.1016/j.jbc.2023.104692)
Supplement: Supporting Table S1 and Figures S1–S6 [file mmc1.docx]

Supporting Information
**Enzymatic comparison of two homologous enzymes reveals N-terminal domain of chondroitinase ABC I regulates substrate selection and product generation**

Min Du^1^, Lin Wei^1^, Min Yuan^1^, Ruyi Zou^1^, Yingying Xu^1^, Xu Wang^1^, Wenshuang Wang^1,*^ and Fuchuan Li^1, 2,*^

**Author’s Affiliation**

^1^National Glycoengineering Research Center and Shandong Provincial Key Laboratory of Carbohydrate Chemistry and Glycobiology, Shandong University;

^2^ College of Marine Life Sciences, Ocean University of China, Qingdao, People’s Republic of China.

**Corresponding author**

*(F. Li) Email: fuchuanli@sdu.edu.cn. Tel: +86-532-58631406 Fax: +86-532-58631405.

*(W. Wang) Email: wenshuangwang@sdu.edu.cn. Tel: +86-532-58631406 Fax: +86-532-58631405.

**Supplemental** **Figures and Tables**

**Table S1.** The disaccharide compositions of CS/DS used in this study.

|  | CS-A | CS-C | CS-E | DS |
| --- | --- | --- | --- | --- |
| Unsaturated disaccharides | 2-AB labeled disaccharide (percentage) mol% | | | |
| Δ^4,5^HexA1-3GalNAc | 1.10 | ND^a^ | 7.10 | 2.40 |
| Δ^4,5^HexA1-3GalNAc(4S) | 80.87 | 23.75 | 35.80 | 86.02 |
| Δ^4,5^HexA1-3GalNAc(6S) | 18.03 | 56.03 | 12.60 | ND |
| Δ^4,5^HexA(2S)1-3GalNAc(4S) | ND | ND | ND | 11.58 |
| Δ^4,5^HexA(2S)1-3GalNAc(6S) | ND | 20.22 | ND | ND |
| Δ^4,5^HexA1-3GalNAc(4S,6S) | ND | ND | 44.50 | ND |
| source | [(43)](https://sciwheel.com/work/citation?ids=14066542&pre=&suf=&sa=0&dbf=0) | this study | [(44)](https://sciwheel.com/work/citation?ids=14066546&pre=&suf=&sa=0&dbf=0) | this study |

*^a^* ND means not detected. ΔHexUA, unsaturated hexuronic acid; GalNAc, *N*-acetyl-galactosamine; 2S, 2-*O*-sulfate; 4S, 4-*O*-sulfate; 6S, 6-*O*-sulfate.


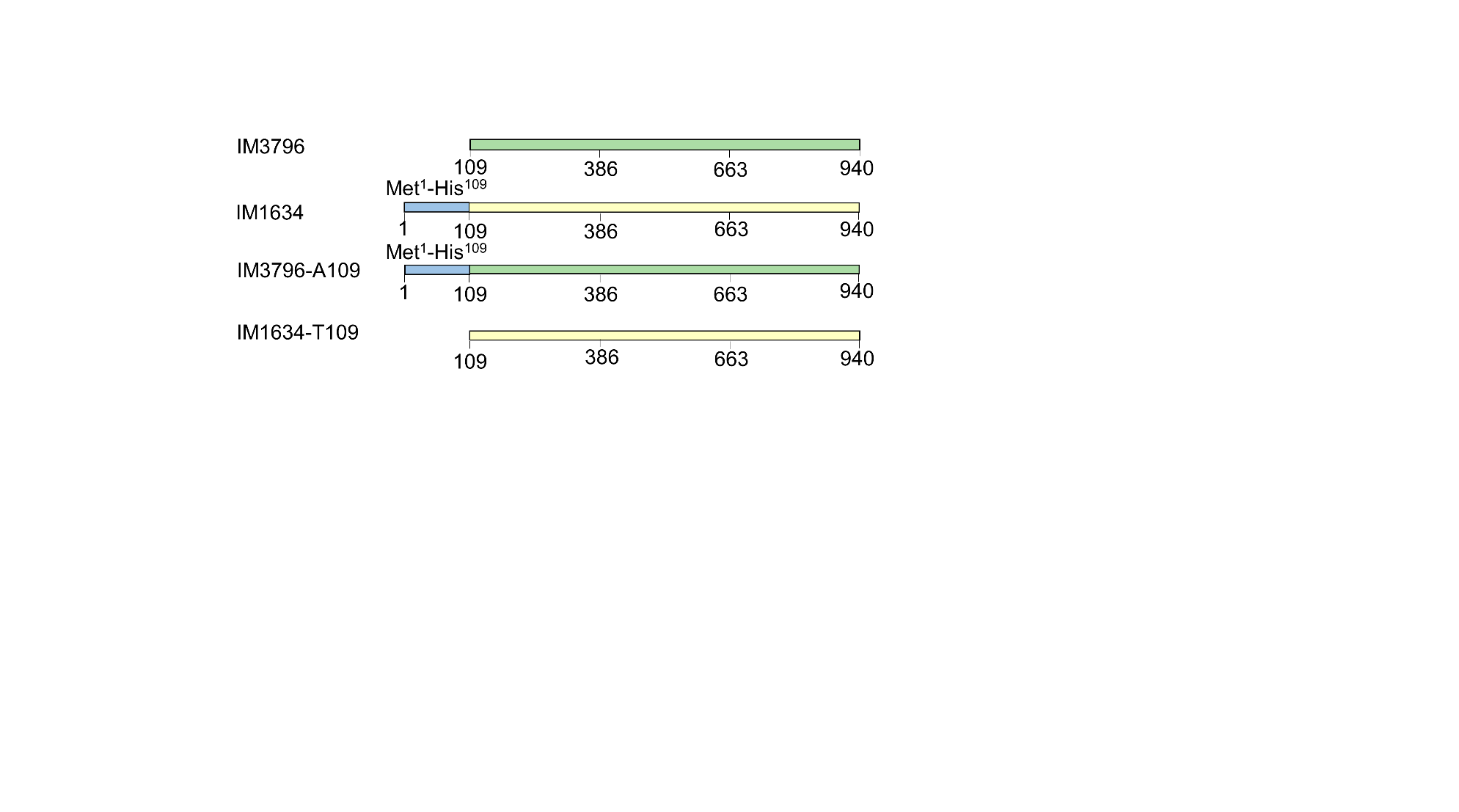


**Figure S1.** Schematic diagram of IM3796, IM1634 and their mutations. IM1634-T109, a deviant of IM1634 by deleting the N-terminal Met^1^-His^109^; IM3796-A109, a deviant of IM3796 by adding the N-terminal Met^1^-His^109^ from IM1634. The numbers represent the number of amino acids.





**Figure S2.** Substrate specificity of IM3796 and IM1634. Various polysaccharides (150 μg), including HA, CS-A, CS-C, CS-E, DS, alginate, Hep and HS, were treated with IM3796 (10 μM, 15 μl) for 2h or IM1634 (9 μM, 2 μl) for 10 min at 30 °C in the 50 mM NaH_2_PO_4_-Na_2_HPO_4_ buffer (pH 7.0). The degradation velocities were determined by measuring the absorbance at 232 nm. Data are shown as the percentage of the activity obtained from the optimum substrate.


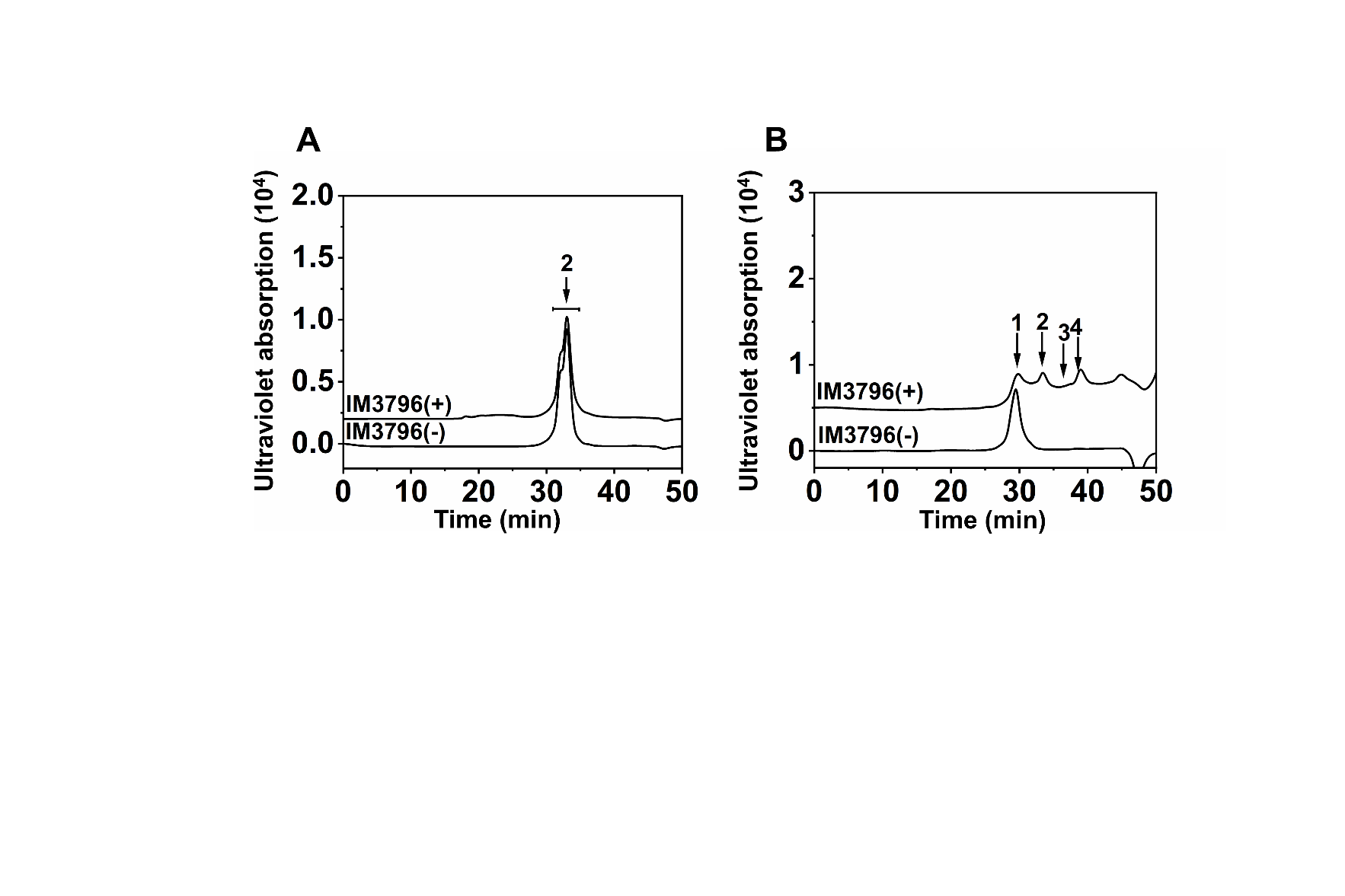


**Figure S3.** Minimum substrate analysis of IM3796. The size-defined tetrasaccharides (A) and hexasaccharides (B) prepared from CS-C by commercial CSase ABC were exhaustively digested without or with IM3796 under the optimal conditions for 48 h. The products were analyzed by gel filtration chromatography on a Superdex Peptide 10/300 GL column with a UV detector at 232 nm. The elution positions of the following standard oligosaccharides are indicated by arrows: 1, unsaturated hexasaccharides; 2, unsaturated tetrasaccharides; 3, unsaturated disulfated disaccharides; 4, unsaturated monosulfated disaccharides.


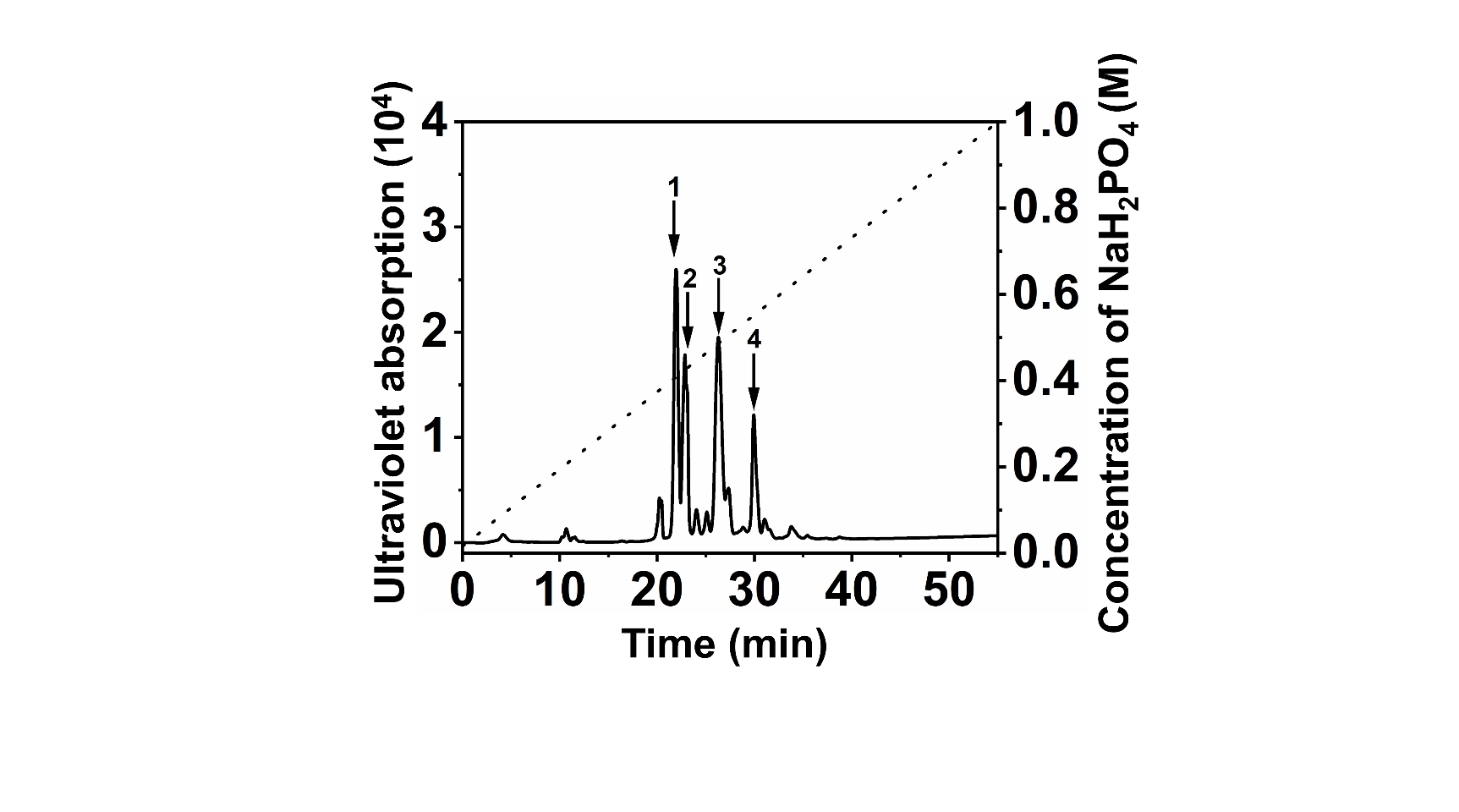


**Figure S4.** Subfractionation of tetrasaccharides from the exhaustive digestion of CS-C by IM3796. The size-defined tetrasaccharides isolated from the final digest of CS-C by IM3796 were subfractionated by anion exchange HPLC on a YMC-Pack PA-G column eluted by a linear gradient from 16 mM to 1 M NaH_2_PO_4_ buffer (shown by the dotted line) during a 60 min period, and the major subfractions (Fr.1-4) were collected under detection by a UV detector at 232 nm.


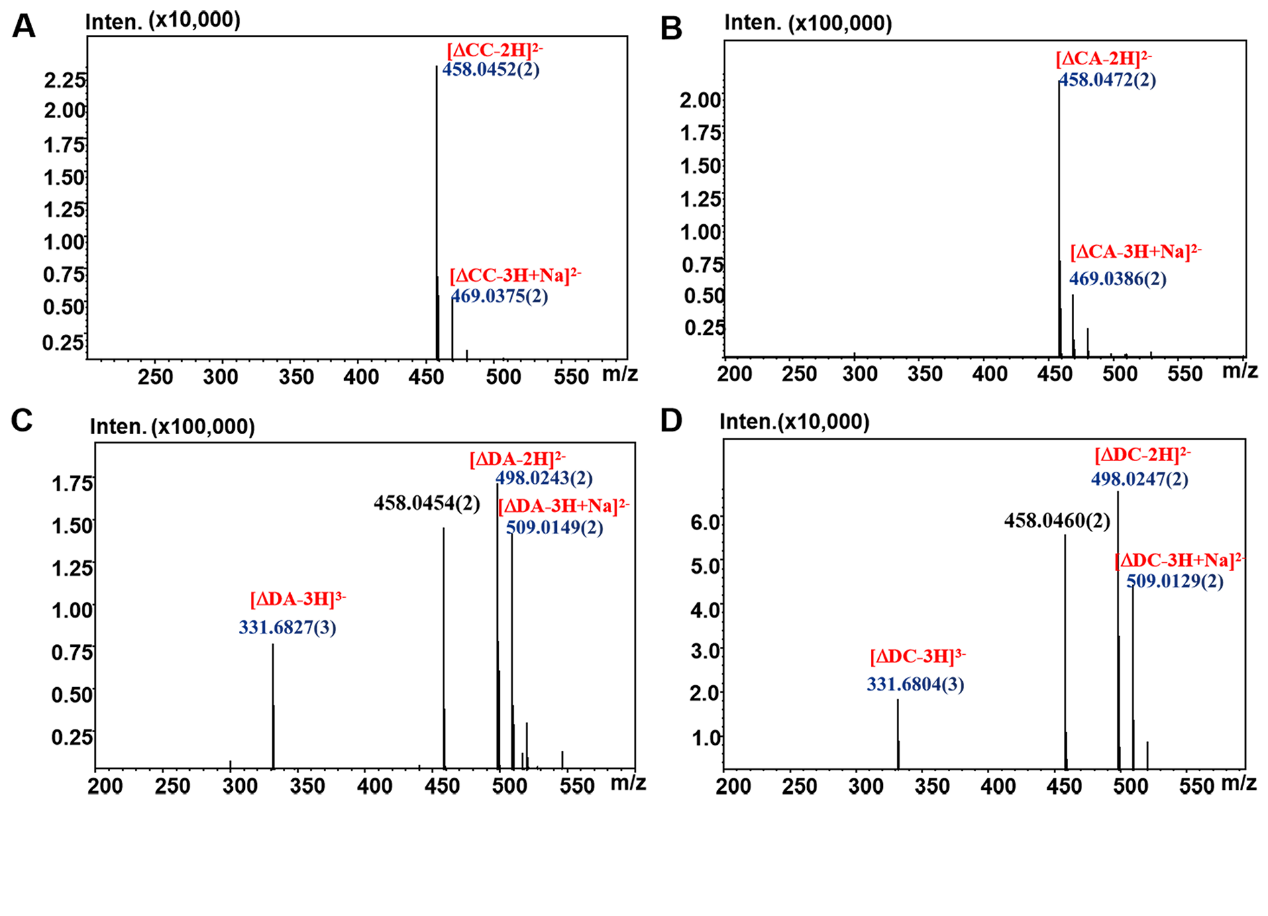
**Figure S5.** Time-of-flight mass spectra analysis of the tetrasaccharide subfractions (Fr.1-4). The molecular masses of four major tetrasaccharide subfractions (Fr.1-4) from the final product of CS-C digested by IM3796 were confirmed by ESI-MS on an IT-TOF (LCMS-IT-TOF) hybrid mass spectrometer. ΔC-C (A), ΔC-A (B), ΔD-A (C) and ΔD-C (D). Inten., intensity. The signals m/z 458.0454(2) and m/z 458.0460(2) in C and D should be due to the desulfurization of trisulfated tetrasaccharide during negative electrospray ionization mass spectrometry [(53, 54)](https://sciwheel.com/work/citation?ids=14096519,13547743&pre=&pre=&suf=&suf=&sa=0,0&dbf=0&dbf=0).


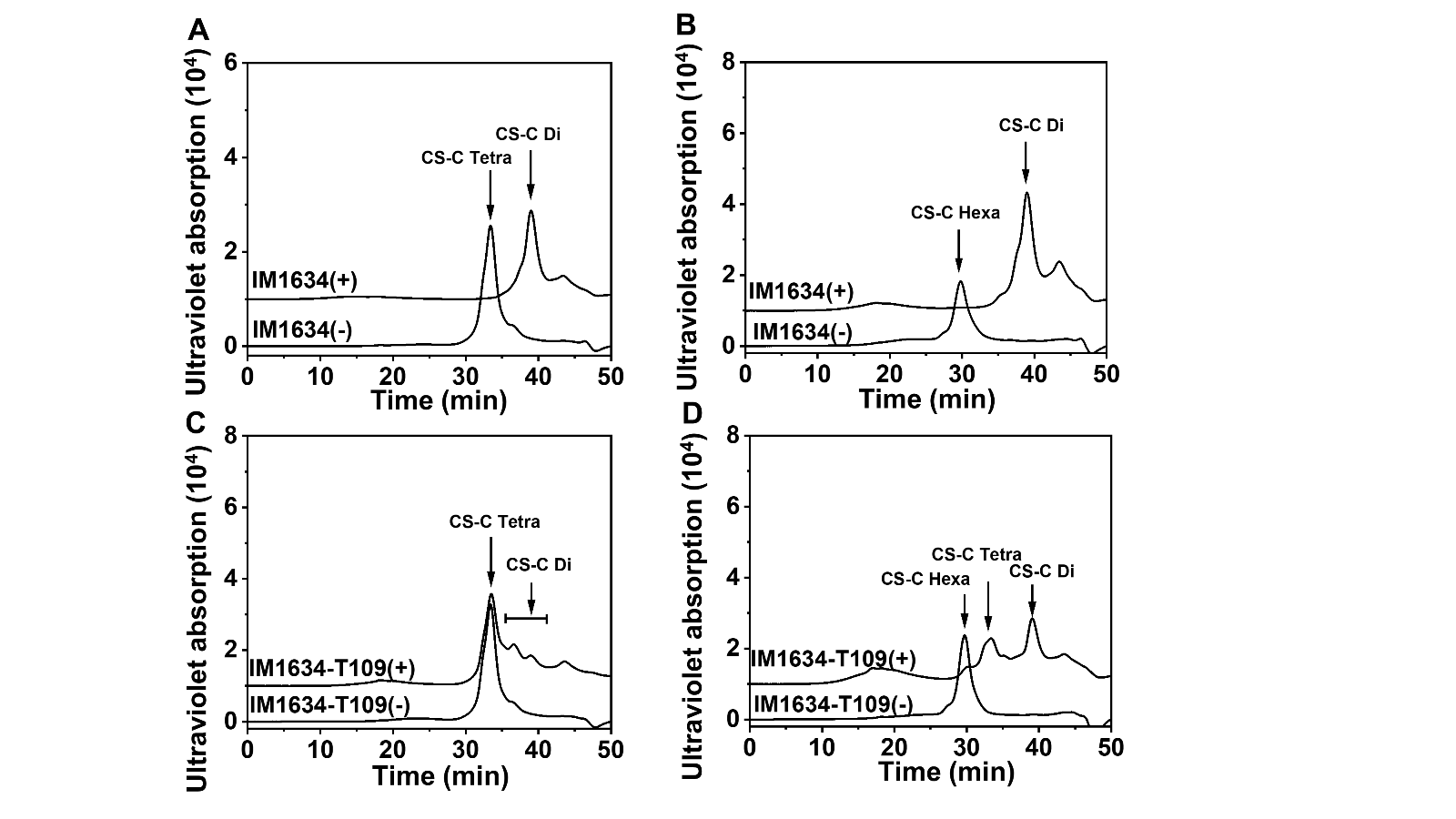


**Fig. S6.** Resultant analysis of CS-C tetrasaccharide or hexasaccharide treated by IM1634 and IM1634-T109. The tetra- or hexasaccharide fraction prepared from the partial digest of CS-C by commercial CSase ABC was exhaustively treated without or with IM1634 and its truncated variant IM1634-T109, respectively. The resultants were analyzed by gel filtration using a SuperdexTM Peptide 10/300 GL column (GE Healthcare) monitored at 232 nm by a UV detector. Di, disaccharide; Tetra, tetrasaccharide; and Hexa, hexasaccharide.

**References**

[43. Xu, Y., Shi, L., Qin, Y., Yuan, X., Wang, X., Zhang, Q., Wei, L., Du, M., Liu, Y., Yuan, M., Xu, X., Cheng, R., Zou, R., Wang, W., and Li, F. (2022) A mutated glycosaminoglycan-binding domain functions as a novel probe to selectively target heparin-like epitopes on tumor cells. *J. Biol. Chem.* **298**, 102609](https://sciwheel.com/work/bibliography/14066542)

[44. Peng, C., Wang, Q., Jiao, R., Xu, Y., Han, N., Wang, W., Zhu, C., and Li, F. (2021) A novel chondroitin sulfate E from *Dosidicus gigas* cartilage and its antitumor metastatic activity. *Carbohydr. Polym.* **262**, 117971](https://sciwheel.com/work/bibliography/14066546)

[53. Zaia, J., and Costello, C. E. (2003) Tandem mass spectrometry of sulfated heparin-like glycosaminoglycan oligosaccharides. *Anal. Chem.* **75**, 2445–2455](https://sciwheel.com/work/bibliography/14096519)

[54. Kailemia, M. J., Li, L., Ly, M., Linhardt, R. J., and Amster, I. J. (2012) Complete mass spectral characterization of a synthetic ultralow-molecular-weight heparin using collision-induced dissociation. *Anal. Chem.* **84**, 5475–5478](https://sciwheel.com/work/bibliography/13547743)
